# Prevalence, Years Lived With Disability, and Time Trends for 16 Causes of Blindness and Vision Impairment: Findings Highlight Retinopathy of Prematurity

**DOI:** 10.3389/fped.2022.735335

**Published:** 2022-03-11

**Authors:** Rui-Heng Zhang, Yue-Ming Liu, Li Dong, He-Yan Li, Yi-Fan Li, Wen-Da Zhou, Hao-Tian Wu, Ya-Xing Wang, Wen-Bin Wei

**Affiliations:** ^1^Beijing Key Laboratory of Intraocular Tumor Diagnosis and Treatment, Beijing Ophthalmology and Visual Sciences Key Lab, Medical Artificial Intelligence Research and Verification Key Laboratory of the Ministry of Industry and Information Technology, Beijing Tongren Eye Center, Beijing Tongren Hospital, Capital Medical University, Beijing, China; ^2^Beijing Institute of Ophthalmology and Beijing Ophthalmology and Visual Science Key Lab, Beijing Tongren Eye Center, Beijing Tongren Hospital, Capital Medical University, Beijing, China

**Keywords:** prevalence, years lived with disability, blindness, visual impairment, retinopathy of prematurity

## Abstract

**Background:**

Cause-specific prevalence data of vision loss and blindness is fundamental for making public health policies and is essential for prioritizing scientific advances and industry research.

**Methods:**

Cause-specific vision loss data from the Global Health Data Exchange was used. The burden of vision loss was measured by prevalence and years lived with disability (YLDs).

**Findings:**

In 2019, uncorrected refractory error and cataract were the most common causes for vision loss and blindness globally. Women have higher rates of cataract, age-related macular degeneration (AMD), and diabetic retinopathy (DR) than men. In the past 30 years, the prevalence of moderate/severe vision loss and blindness due to neonatal disorders has increased by 13.73 and 33.53%, respectively. Retinopathy of prematurity (ROP) is the major cause of neonatal disorders related vision loss. In 2019, ROP caused 101.6 thousand [95% uncertainty intervals (UI) 77.5–128.2] cases of vision impairment, including 49.1 thousand (95% UI 28.1–75.1) moderate vision loss, 27.5 thousand (95% UI 19.3–36.60) severe vision loss and, 25.0 thousand (95% UI 14.6–35.8) blindness. The prevalence of new-onset ROP in Africa and East Asia was significantly higher than other regions. Variation of preterm birth prevalence can explain 49.8% geometry variation of ROP-related vision loss burden among 204 countries and territories. After adjusting for preterm prevalence, government health spending per total health spending (%), rather than total health spending per person, was associated with a reduced burden of ROP-related vision loss in 2019 (−0.19 YLDs for 10% increment). By 2050, prevalence of moderate, severe vision loss and blindness due to ROP is expected to reach 43.6 (95% UI 35.1–52.0), 23.2 (95% UI 19.4–27.1), 31.9 (95% UI 29.7–34.1) per 100,000 population.

**Conclusion:**

The global burden of vision loss and blindness highlights the prevalent of ROP, a major and avoidable cause for childhood vision loss. Advanced screening techniques and treatments have shown to be effective in preventing ROP-related vision loss and are urgently needed in regions with high ROP-related blindness rates, including Africa and East Asia.

## Introduction

Vision loss is a major cause of functional impairment globally, greatly decreasing life quality and increasing social burden. Recently, two comprehensive reports on the burden of vision loss were published by GBD and The Vision Loss Expert Group (1, 2). It is estimated that 43.3 million [95% uncertainty intervals (UI) 37.6–48.4] people were blind globally, and 295 million (95% UI: 267–325) people had moderate and severe vision loss in 2020 (1). The leading causes globally for vision loss are uncorrected refractive error and cataract (2, 3). Other vision-threatening conditions include age-related macular degeneration (AMD), glaucoma, and diabetic retinopathy (DR). Communicable diseases, such as trachoma and onchocerciasis, are still major communicable causes for vision loss in underdeveloped countries and areas (4, 5). Besides, because of the rapid advance of neonatal care, retinopathy of prematurity (ROP) and other neonatal disorders are now the most common causes of vision loss in children (6, 7). Despite national programs of vitamin A supplementation has implemented in 82 countries, vitamin A deficiency remains prevalent in south Asia and sub-Saharan Africa (8, 9).

Cause-specific prevalence data of distance vision loss and blindness is fundamental for making public health policies and is essential for prioritizing scientific advances and industry research. Previous studies have focused on primary blinding eye disease, including glaucoma, cataract, AMD, DR, and refractive error. Less attention has been paid to minor causes, such as neonatal disorders and nutritional deficiencies. These causes are the major contributions to the vision loss burden among children and adolescents (7).

The present study aimed to quantify the vision loss estimates due to 16 kinds of diseases from 1990 to 2019 using the Global Burden of Diseases 2019 (GBD 2019). Based on the current epidemiological situation, we mainly explore the current disease burden, associated factors, and future ROP-related vision loss burden.

## Method

### Data Source

The GBD 2019 Study provided Cause-specific vision loss data in the Global Health Data Exchange (http://ghdx.healthdata.org/gbd-results-tool, accessed on December 15, 2020). We included distance vision loss and blindness data from GBD dataset. Details of the study's methodology have been described previously (6). In brief, The Vision Loss Expert Group has systematically reviewed the scientific literature published between 1980 and 2018 by commissioning the York Health Economics Consortium, UK, to search Embase, SciELO, MEDLINE, WHOLIS, and Open Gray, and additional gray literature sources (2). Bayesian meta-regression tool synthesized all available data, adjusting for different case definitions or sampling strategies. Distance vision loss was divided into three categories: moderate vision impairment (defined as visual acuity of ≥6/60 and <6/18), severe vision impairment (visual acuity of ≥3/60 and <6/60), and blindness (visual acuity of <3/60 or <10° visual field around central fixation). Then, location, year, age, and sex-specific estimates of vision loss and blindness were calculated using a wide range of standardized analytical procedures, including data screening, data adjustment, DisMod-MR 2.1 modeling.

### Outcomes and Related Factors

The primary outcomes of the present study were the total number of cases in the population (Number), total cases per 100,000 population (Rate), age-standardized cases per 100,000 population (age-standardized rate), and years lived with disability (YLDs) due to various causes. YLDs were calculated by multiplying the prevalence of the eye disease and its associated disability weight in each age-sex-country-year population. The disability weight represents the magnitude of health loss associated with the outcome. It was measured on a scale from 0 to 1, where 0 implied a state equivalent to full health, and 1 was equivalent to death (10). Age-standardization was computed using a standard population age structure updated in each GBD round. Currently, the standard population was taken as the average of age-specific distributions (non-weighted) from GBD 2019 population estimates for countries with at least 5 million people in the year 2019 (11).

A total of 16 causes of vision loss were included in the analysis: communicable diseases (meningitis, encephalitis, onchocerciasis, trachoma, malaria), neonatal disorders (retinopathy of prematurity, neonatal sepsis and other neonatal infections, hemolytic disease, and other neonatal jaundice, neonatal encephalopathy due to birth asphyxia and trauma), nutritional deficiency (vitamin A deficiency), glaucoma, cataract, AMD, DR, refractive error, and a residual category of other vision loss (Supplementary Table 1).

We analyzed the relationship between temporal trends of vision impairment burden and socio-demographic index (SDI). SDI is a summary measurement constructed based on the geometric mean of income per capita, average years of schooling among people aged 15 years or older, and the total fertility rate. It quantitates a country or territory's level of socio-demographic development (12). Then SDIs were transformed into quintiles for analysis (low-SDI, low-middle-SDI, middle-SDI, high-middle-SDI, or high-SDI). For health spending estimates, four financing sources were included in the analysis: government, out-of-pocket, and prepaid private health spending, which collectively makes up domestic health spending; and development assistance for health, which includes international disbursements for health low-income and middle-income countries (13).

### Forecasting ROP-Related Vision Loss Burden to 2050

Auto-Regressive Integrated Moving Average (ARIMA) model predict future ROP-related vision loss burden. In ARIMA (*p, d, q*) model, *p* represents the number of lag observations; *d* represents the number of times input raw data are different to make the model stationary; *q* represents the size of moving average window applied to lagged observations. It was performed on Stats package (version 4.1.1) to forecast the health burden caused by ROP in terms of age-standardized prevalence rates from 2020 to 2050.

### Statistics Analysis

Linear regression was used to estimate associated factors of ROP disease burden. All analysis was performed in Stata version 15.0 (StataCorp LLC, College Station, TX, USA) and R Statistical Software (version 4.0.3; R Foundation for Statistical Computing, Vienna, Austria).

## Results

### The Burden for Vision Loss and Blindness in 2019

Regional differences were found in the burden of vision impairment in 2019. We estimated that Southeast Asia, South Asia, North Africa, Middle East, Ease and West Sub-Saharan Africa, Tropical and Andean Latin America remained the primary area with high age-standardized YLDs for all-cause distance vision loss and blindness. To explore regional differences, the predominant causes for vision loss in 204 countries and territories were plotted (Supplementary Tables 2–4; Supplementary Figures 1–18). Cataract and uncorrected refractive error have become the most common causes for vision impairment in most countries and territories.

The burden of distance vision loss and blindness was also different between age and sex. As exhibited in Supplementary Figure 19, we observed a strong association between age and vision impairment. To further elaborate on the sex differences in all 16 causes of vision impairment, we generated cause-YLD ratios by female YLD rates divided by male YLD rates (Supplementary Figure 20). Among sex groups, glaucoma [11 cases (95% UI 7–16) per 100,000 males vs. 10 (7–15) per 100,000 females] has a higher YLD rates in men, while cataract [77 (95% UI 54–108) per 100,000 males vs. 113 (79–157) per 100,000 females], AMD [6.4 (95% UI 4.3–9.2) per 100,000 males vs. 9.8 (6.6–13.9) per 100,000 females], and DR [4.1 (95% UI 2.6–5.8) per 100,000 males vs. 5.3 (3.5–7.6) per 100,000 females] have higher YLD rates in women. We did not find a gender difference in neonatal disorders (including ROP) or vitamin A deficiency in children.

### Change of Burden for Vision Loss in 1990–2019 Highlights Neonatal Disorders

**Figure 1:**
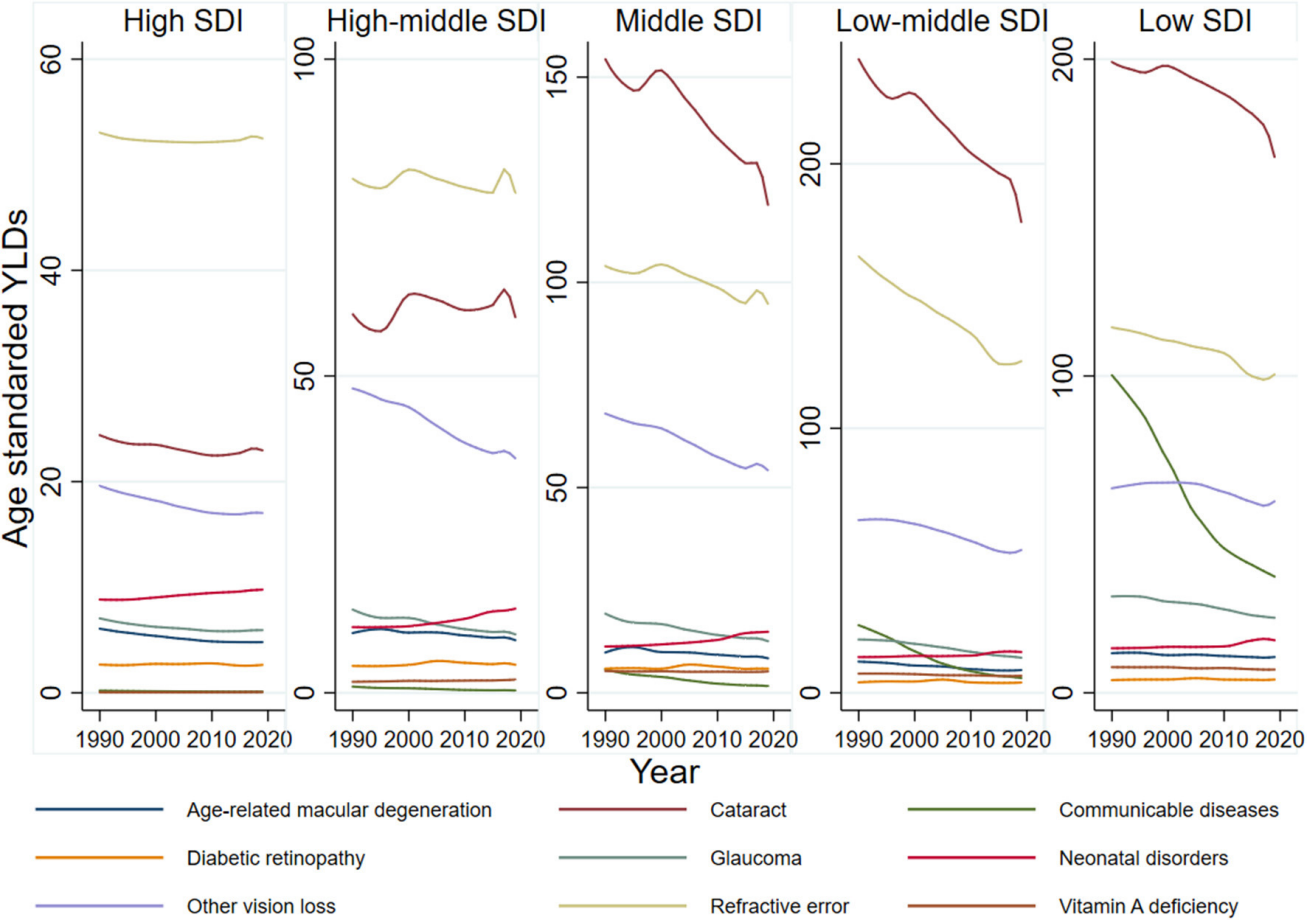
Age-standardized, cause-specific YLD rate of blindness and distance vision impairment by Socio-Demographic Index groups, 1990–2019.

**Figure 2:**
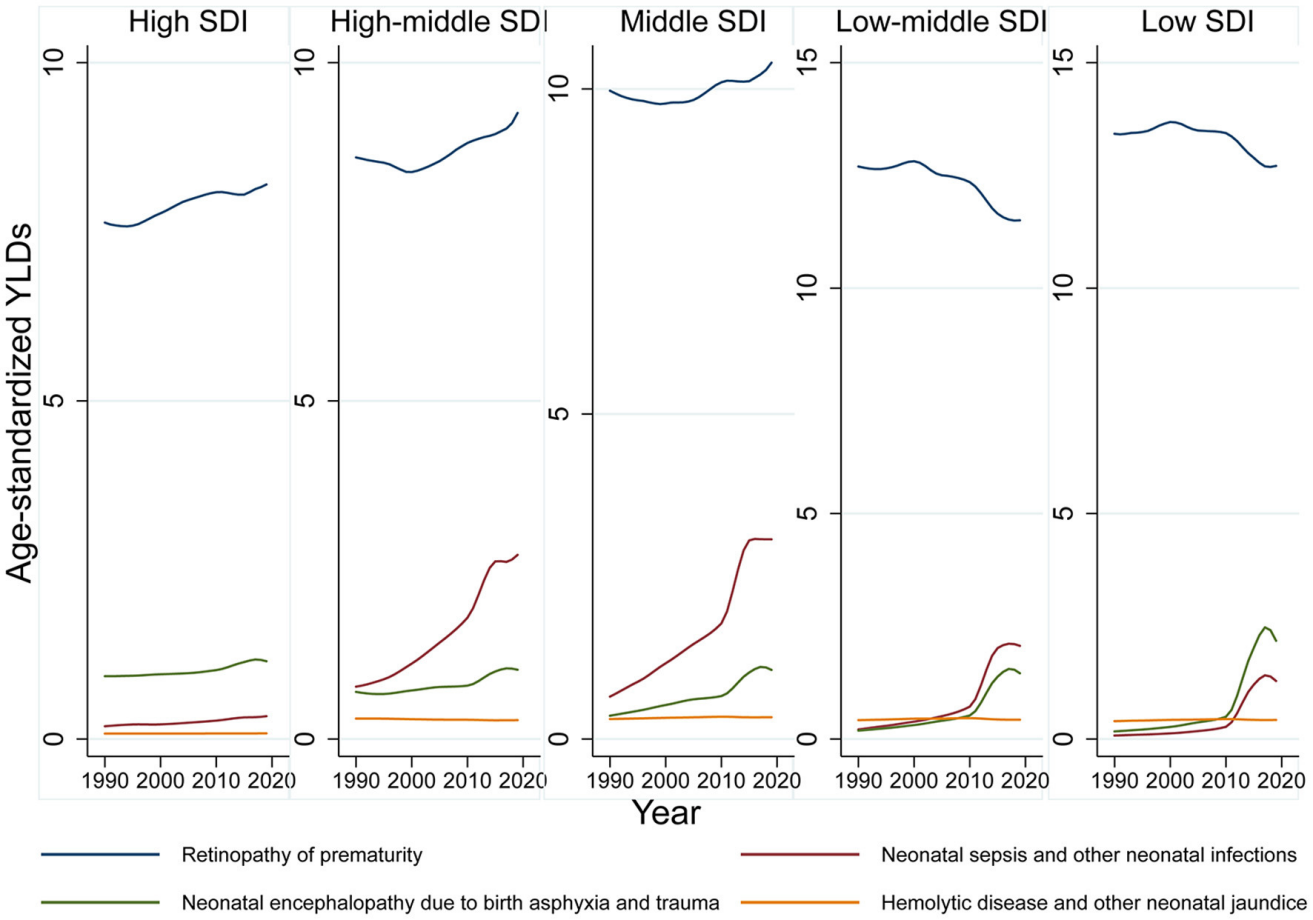
Age-standardized, cause-specific YLD rate due to neonatal disorders.

Globally, age-standardized blindness prevalence decreased by −27.14% from 1990 to 2019. Although total cases of moderate, severe vision loss and blindness increased, age-standardized prevalence decreased or remained stable, which indicated population growth and aging contributed to increased total cases (Supplementary Table 5). YLDs trend across SDI quintiles revealed refractive error was the major vision-threatening condition in high and high-middle SDI countries. Cataract was the major cause in middle, middle-low, and low SDI countries, and it continued to decline alongside refractive error during this period. Vision loss due to communicable diseases, the third leading cause for vision impairment in low SDI countries, has rapidly declined in last 30 years (Supplementary Figure 21). In contrast there has been an increasing diseases burden of neonatal disorders related vision loss during the past 30 years (Figure 1). From 1990 to 2019, prevalence of moderate/severe vision impairment and blindness due to neonatal disorders has increased by 13.73 and 33.53%, respectively. ROP is the major cause of neonatal disorders related vision loss. By 2019, the prevalence of moderate, severe vision loss and blindness due to ROP reached 35 (95% UI 20–53), 20 (95% UI 14–26), and 32 (95% UI 24–40) (Figure 2).

### The Global Burden and Trend of ROP-Related Vision Loss Burden

**Figure 3:**
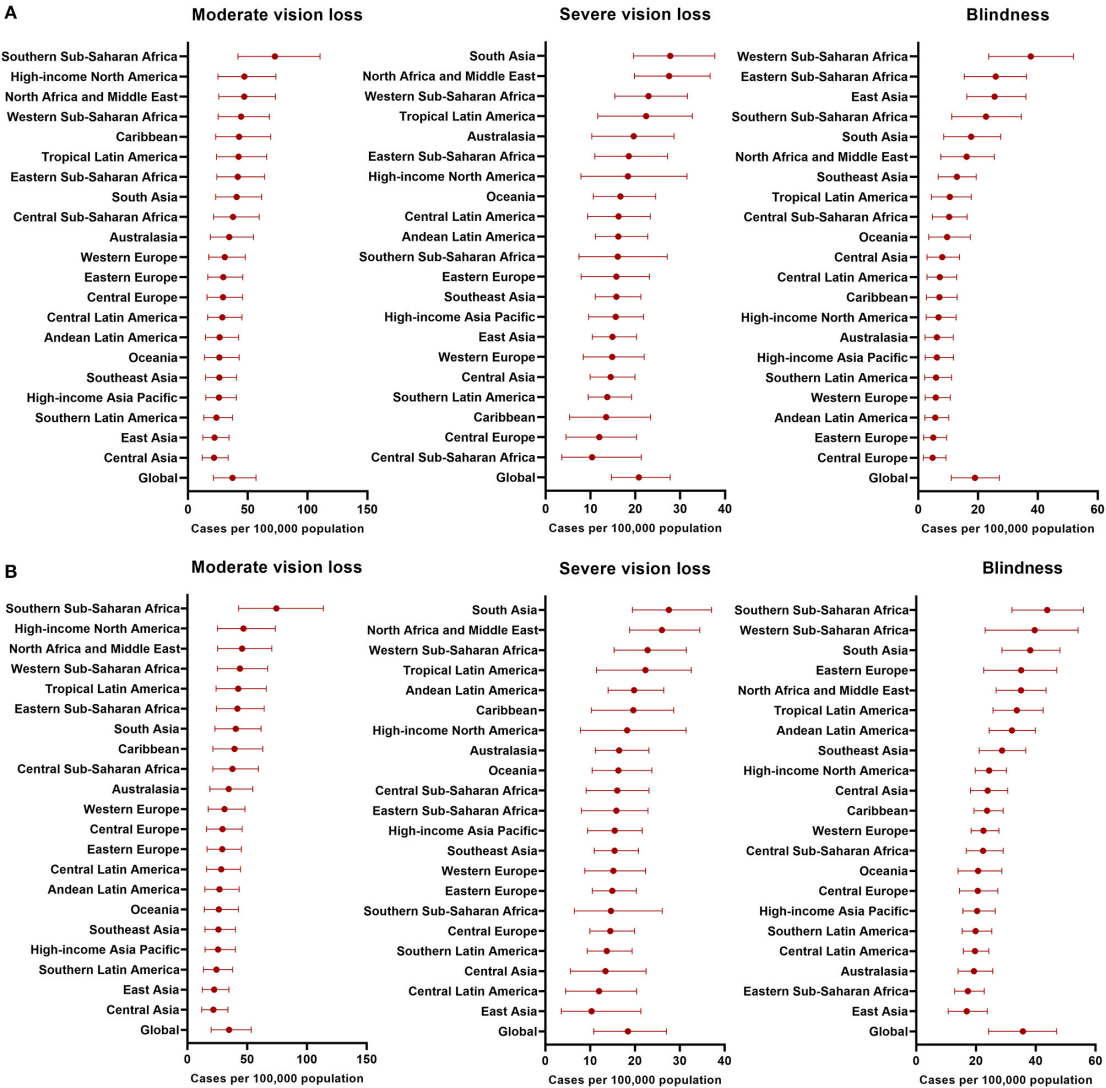
Incidence and Prevalence of ROP-related blinding varies among 21 GBD regions. **(A)** Incidence of ROP in 2019; **(B)** Prevalence of ROP in 2019.

For all age groups combined, it is estimated that 15.2 million (95% UI 15.1–15.3) preterm babies, with 9.4% (95% UI 7.7–11.4) complicated with any stage of ROP that causes moderate/severe vision loss or blindness. The prevalence of ROP-related blinding varies among 21 GBD regions, with the highest of 44 (95% UI 32–56) in Southern Sub-Saharan Africa and the lowest of 17 (95% UI 11–24) cases per 100,000 population in East Asia (Figure 3). The prevalence of ROP among all infants<1-year-old reflects the annual incidence of ROP. In 2019, new-onset ROP caused 101.6 thousand (95% UI 77.5–128.2) cases of vision impairment, including 49.1 thousand (95% UI 28.1–75.1) moderate vision loss, 27.5 thousand (95% UI 19.3–36.60) severe vision loss, and 25.0 thousand (95% UI 14.6–35.8) blindness. Especially, the incidence of new-onset ROP-related blindness was still higher in Sub-Saharan Africa and Asia than other regions.

**Figure 4:**
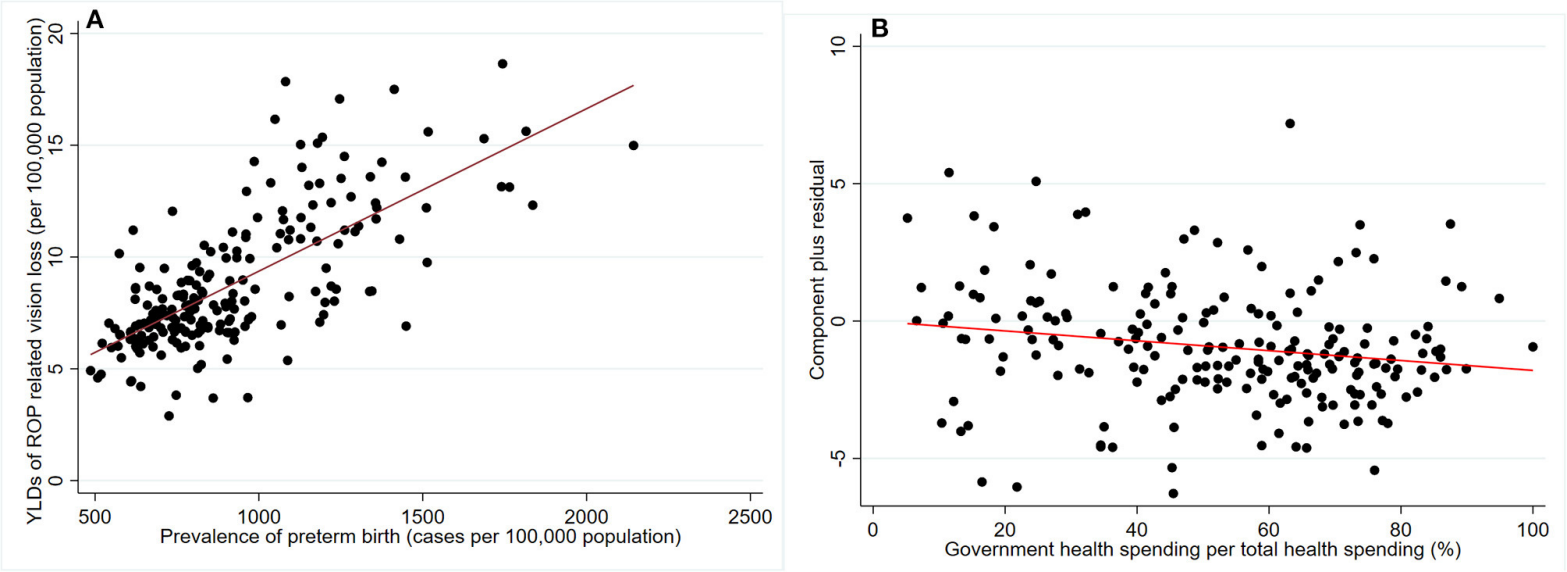
Scatter plot and linear prediction of YLDs related to retinopathy of prematurity. **(A)** association between preterm birth prevalence and ROP-related vision loss burden among 204 countries and territories; **(B)** association between government health spending per total health spending (%) and ROP-related vision loss burden among 204 countries and territories, adjusted for preterm birth prevalence.

Variation of preterm birth prevalence can explain 49.8% geometry variation of ROP-related vision loss burden among 204 countries and territories (Figure 4A). Health expenditure and social development were expected to be closely related to preterm birth prevalence. Cross-sectional analysis revealed that total healthy spending per person (−39.83 cases/100,000 population for 1,000 USD increment) and socio-demographic index (−28.75 cases/100,000 population for 0.1 increment) was significantly associated with preterm birth prevalence (Table 1). As for financing sources of total health spending, government health spending out-of-pocket health spending per person were negatively associated with the preterm birth prevalence. Development assistance for health per person was positively associated with the preterm birth prevalence, reflecting deficient total health spending. After adjusting for preterm prevalence, only government health spending per total health spending was associated with a reduced burden of ROP-related vision loss in 2019 (−0.19 YLDs for 10% increment, Table 1 and Figure 4B). This association remained significant after adjusting for both preterm prevalence and total health spending per person [−0.22 YLDs for 10% increment, 95% confidence interval (CI) −0.38 to −0.06, *P* = 0.006].

**Table 1:** Association between Health spending with burden of ROP-related vision loss and preterm prevalence among 204 countries and territories in 2019.

	**ROP-related vision loss (YLDs)** ^**†**^		**Preterm birth prevalence**	
	**Coefficient**	**95% Confidence interval**	**Coefficient**	**95% Confidence interval**
Socio-demographic index (per 0.1 increment)	−0.01	[−0.28, 0.10]	–**78.09**	**[**–**98.84**, –**57.35]**
Total health spending per person (per 1,000 USD increment)	−0.06	[−0.23, 0.10]	–**39.84**	**[**–**61.01**, –**18.67]**
Total health spending per person (per 1,000 PPP increment)	−0.01	[−0.18, 0.16]	–**47.39**	**[**–**68.28**, –**26.50]**
Government health spending per person (per 1,000 USD increment)	−0.07	[−0.31, 0.17]	**−63.11**	**[−93.23**, **−33.00]**
Government health spending per person (per 1,000 PPP increment)	−0.02	[−0.25, 0.22]	**−69.34**	**[−97.98**, **−40.69]**
Prepaid private health spending per person (per 1,000 USD increment)	−0.20	[−0.74, 0.34]	−13.09	[−87.31, 61.13]
Prepaid private health spending per person (per 1,000 PPP increment)	−0.10	[−0.72, 0.51]	−7.35	[−91.67, 76.97]
Out-of-pocket health spending per person (per 1,000 USD increment)	−0.24	[−1.23, 0.75]	**−283.34**	**[−406.59**, **−160.09]**
Out-of-pocket health spending per person (per 1,000 PPP increment)	0.28	[−0.65, 1.21]	**−318.02**	**[−427.18**, **−208.85]**
DAH per person (per 1,000 USD increment)	−11.36	[−26.05, 1.33]	863.01	[−1015.49, 2741.67]
DAH per person (per 1,000 PPP increment)	−4.81	[−15.16, 5.55]	**2124.18**	**[769.89, 3478.47]**
Government health spending per total health spending (per 10% increment)	**−0.19**	**[−0.33**, **−0.04]**	**−51.27**	**[−68.29**, **−34.26]**
Total health spending per GDP	−0.03	[−0.13, 0.07]	**−14.62**	**[−27.56**, **−1.68]**
Government health spending per GDP	−0.10	[−0.22, 0.02]	**−38.71**	**[−53.59**, **−23.83]**

*PPP, Purchasing power parities; DAH, Development assistance for health. Preterm birth prevalence was measured as cases per 100,000 population*.

† *Adjusted for Preterm birth prevalence in 2019. Bold value indicates P < 0.05*.

**Figure 5:**
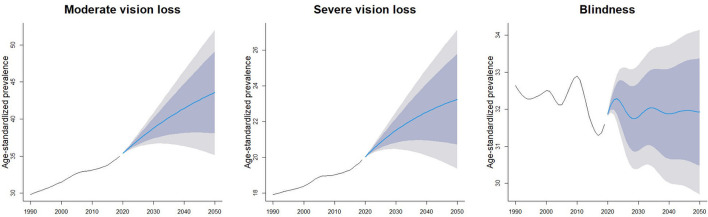
Forecast of age-standardized prevalence per 100,000 population due to retinopathy of prematurity.

During the past 30 years, the ROP-related vision loss burden has increased in high, high-middle, middle SDI countries, and decreased in low-middle, low SDI countries. By 2050, global prevalence of moderate, severe vision loss and blindness due to ROP is expected to reach 43.6 (95% UI 35.1–52.0), 23.2 (95% UI 19.4–27.1), 31.9 (95% UI 29.7–34.1) per 100,000 population (Figure 5).

## Discussion

This study found that cataract and uncorrected refractive error remains the most common causes for distance vision loss and blindness. Moreover, the gender difference was found in the disease burden of vision loss. Women have higher rates of cataract, AMD, and DR than men. In the past 30 years, there has been an increasing diseases burden of neonatal disorders related vision loss. ROP is the primary cause of neonatal disorders related vision loss. Without more effective screening and interventions strategy, the burden of ROP-related vision loss was expected to increase in the future.

Avoidable visual impairments constitute major burden of vision loss globally. We found that age-standardized YLDs for cataract have declined rapidly in middle SDI, low-middle SDI, and low SDI counties, indicating the improved efficiency of ophthalmic screening and the quality of treatment delivered in these areas. However, the residual YLDs indicated that it had not been fully addressed in most world regions, including high-incomes and high-SDI regions. Further efforts need to promote the accessibility of high-quality cataract surgery, especially in developing countries and remote areas. Further initiatives, programs, or mass campaigns are needed to develop infrastructure, personnel, and economic strategies to provide sustainable, high-quality training, counseling, and facility (14). Uncorrected refractive error, including myopia, hyperopia, and astigmatism, is another major cause of vision impairment (15). It is prevalent in Latin America, North Africa, the Middle East, and South Asia and that is leading cause of vision impairment in high and high-middle SDI countries. With decreased outdoor time, increased near-work activities, and excessive use of near electronic devices and other factors, myopia and high myopia have been anticipated to significantly increase (16, 17). It is estimated that 5.2% of the global population will have high myopia in 2020 and will significantly increase to 9.8% of the global population in 2050 (18). Myopia brings further vision challenges because high myopia increases the risk of pathologic ocular changes such as glaucoma, retinal detachment, and myopic maculopathy, all of which can cause irreversible vision loss (19). Because effective interventions for myopic maculopathy are still limited, future researches need to focus on preventing or delaying myopia onset and retarding myopia progression through lifestyle modifications and medical interventions.

In 2019, ROP caused 49.1 thousand, 27.5 thousand, and 25.0 thousand moderate, severe vision loss and blindness cases, respectively. The increasing diseases burden of ROP-related vision loss may be caused by the rapid advance of neonatal care that improves preterm infants' survival (20, 21). As advance of ROP screening and treatments in the past 30 years (22), the blindness rate due to ROP sightly decreased by 3.23%. However, we observed a significant disparity between prevalence and new-onset ROP-related blindness incidence. The developed regions, such as Europe and North America, exhibited the “high prevalence and low incidence,” whereas the developing regions, such as Africa and East Asia, exhibited the “high prevalence and high incidence” of ROP-related blindness incidence. These results highlight that advanced screening techniques and treatments effectively prevent ROP-related blindness but are unevenly distributed. Early diagnosis and timely treatments are urgently needed in these regions.

Compared to cataract and most uncorrected refractive error, progression of glaucoma, AMD, DR, and myopic maculopathy can lead to irreversible vision loss. The characteristics of these diseases and limited options for curing these diseases highlight the screening and effective management once diagnosed. Unfortunately, we still lack precise diagnostic measures for glaucoma in the screening setting, and a large proportion of patients with glaucoma remain undiagnosed in developed, developing, and underdeveloped regions of the world (23). Furthermore, the number of adults with diabetes was expected to surpass 700 million globally (24), and it is estimated that about one-third of people with diabetes will develop DR (25). Moreover, with global population growth and aging, AMD increasingly becomes an important vision-threatening condition in the elderly (26). Artificial intelligence-based screening and referring could tackle these challenges. Nowadays, artificial intelligence is applied for detecting DR, AMD, glaucoma, myopic maculopathy, and papilledema by using multimodality imaging, including fundus photographs, optical coherence tomography (OCT), and fundus fluorescence angiography (FFA) images (27, 28). Novel algorism systems have been developed that are able to identify multiple ocular diseases and lesions (29, 30). It can be anticipated that artificial intelligence would provide automated, immediate feedback in screening settings.

Vitamin A deficiency and communicable diseases used to be two leading causes of vision impairment in underdeveloped counties. Despite the established national vitamin A supplementation programs in 82 countries, vitamin A deficiency remains prevalent in south Asia and Africa (Supplementary Figure 18). With COVID-19 interrupting global nutrition programs, it is entirely possible vision loss due to vitamin A deficiency may become newly resurgent in many countries (31). Among communicable diseases, onchocerciasis is still the most common cause of vision impairment in Central Africa. Although the prevalence of these diseases declines rapidly, national programs are needed to fully eliminate these diseases (32).

This study has several limitations. First, the number of studies and quality of the available data are still limited. We found that “other causes” contributed to 124 (95% UI 105–144) blindness in every 100,000 age-standardized population worldwide. Thus, a major part of the causes of blindness and vision impairment has remained uncovered, such as corneal occupation and eye injury. Second, the burden of vision loss may be underestimated due to lacking precise diagnostic measures and universal diagnosis criteria for glaucoma. Furthermore, Furthermore, SDI was used to measure socio-economic position, but this index could not fully represent level of health care. The application of SDI also ignored the social heterogeneity within countries.

## Conclusions

This study demonstrates the prevalence and time trend of 16 disease-related vision loss from 1990 to 2019, stratified by age, gender, and regions. The efforts to eliminate avoidable vision loss and improve unavoidable vision loss have been suboptimal over the last 30 years, especially in ROP. Advanced screening techniques and treatments are effective in preventing ROP-related blindness and are urgently needed in regions with high ROP-related blindness rates, including Africa and East Asia.

## Data Availability Statement

Publicly available datasets were analyzed in this study. This data can be found here: http://ghdx.healthdata.org/gbd-results-tool.

## Author Contributions

R-HZ, Y-ML, LD, and W-BW: conception, design, provision of study materials or patients, and collection and assembly of data. W-BW: administrative support. R-HZ, LD, Y-ML, H-YL, Y-FL, W-DZ, and H-TW: data analysis and interpretation. All authors manuscript writing and final approval of manuscript.

## Funding

This study was supported by the Capital Health Research and Development of Special (2020-1-2052) and Science and Technology Project of Beijing Municipal Science and Technology Commission (Z201100005520045 and Z181100001818003).

## Conflict of Interest

The authors declare that the research was conducted in the absence of any commercial or financial relationships that could be construed as a potential conflict of interest.

## Publisher's Note

All claims expressed in this article are solely those of the authors and do not necessarily represent those of their affiliated organizations, or those of the publisher, the editors and the reviewers. Any product that may be evaluated in this article, or claim that may be made by its manufacturer, is not guaranteed or endorsed by the publisher.

## References

[1] Bourne R Steinmetz JD Flaxman S Briant PS Taylor HR Resnikoff S. Trends in prevalence of blindness and distance and near vision impairment over 30 years: an analysis for the Global Burden of Disease Study. Lancet Global Health. (2021) 9:e130–43. 10.1016/S2214-109X(20)30425-3 33275950 PMC7820390

[2] Blindness GBD Vision Impairment C Vision Vision Loss Expert Group of the Global Burden of Disease S. Causes of blindness and vision impairment in 2020 and trends over 30 years, and prevalence of avoidable blindness in relation to VISION 2020: the Right to Sight: an analysis for the Global Burden of Disease Study. Lancet Global Health. (2020) 9:E144–60. 10.1016/S2214-109X(20)30489-7 33275949 PMC7820391

[3] Xu T Wang B Liu H Wang H Yin P Dong W. Prevalence and causes of vision loss in China from 1990 to 2019: findings from the Global Burden of Disease Study 2019. Lancet Public Health. (2020) 5:e682–91. 10.1016/S2468-2667(20)30254-1 33271081

[4] Taylor HR Burton MJ Haddad D West S Wright H. Trachoma. Lancet. (2014) 384:2142–52. 10.1016/S0140-6736(13)62182-0 25043452

[5] Tekle AH Zouré HG Noma M Boussinesq M Coffeng LE Stolk WA. Progress towards onchocerciasis elimination in the participating countries of the African Programme for Onchocerciasis Control: epidemiological evaluation results. Inf Dis Poverty. (2016) 5:66. 10.1186/s40249-016-0160-7 27349645 PMC4924267

[6] Diseases GBD Injuries C. Global burden of 369 diseases and injuries in 204 countries and territories, 1990-2019: a systematic analysis for the Global Burden of Disease Study 2019. Lancet. (2020) 396:1204–22. 10.1016/S0140-6736(20)30925-9 33069326 PMC7567026

[7] Solebo AL Teoh L Rahi J. Epidemiology of blindness in children. Arch Dis Child. (2017) 102:853–7. 10.1136/archdischild-2016-310532 28465303

[8] Stevens GA Bennett JE Hennocq Q Lu Y De-Regil LM Rogers L. Trends and mortality effects of vitamin A deficiency in children in 138 low-income and middle-income countries between 1991 and 2013: a pooled analysis of population-based surveys. Lancet Global Health. (2015) 3:e528–36. 10.1016/S2214-109X(15)00039-X 26275329

[9] Wirth JP Petry N Tanumihardjo SA Rogers LM McLean E Greig A. Vitamin A supplementation programs and country-level evidence of vitamin A deficiency. Nutrients. (2017) 9:190. 10.3390/nu9030190 28245571 PMC5372853

[10] Network GBoDC. Global Burden of Disease Study 2019 (GBD 2019) Disability Weights. Seattle, WA: Institute for Health Metrics and Evaluation (IHME) (2020).

[11] Vollset SE Goren E Yuan CW Cao J Smith AE Hsiao T. Fertility, mortality, migration, and population scenarios for 195 countries and territories from 2017 to 2100: a forecasting analysis for the Global Burden of Disease Study. Lancet. (2020) 396:1285–306. 10.1016/S0140-6736(20)30677-2 32679112 PMC7561721

[12] Network GBoDC. Global Burden of Disease Study 2019 (GBD 2019) Socio-Demographic Index (SDI) 1950–2019. Seattle, WA: Institute for Health Metrics and Evaluation (IHME) (2020).

[13] Global Burden of Disease Health Financing Collaborator N. Tracking development assistance for health and for COVID-19: a review of development assistance, government, out-of-pocket, and other private spending on health for 204 countries and territories, 1990-2050. Lancet. (2021) 398:1317–43. 10.1016/S0140-6736(21)01258-7 34562388 PMC8457757

[14] Liu YC Wilkins M Kim T Malyugin B Mehta JS. Cataracts. Lancet. (2017) 390:600–12. 10.1016/S0140-6736(17)30544-5 28242111

[15] Li H-Y Liu Y-M Dong L Zhang R-H Zhou W-D Wu H-T. Global, regional, and national prevalence, disability adjusted life years, and time trends for refraction disorders, 1990-2019: findings from the global burden of disease study 2019. BMC Public Health. (2021) 21:1619. 10.1186/s12889-021-11648-1 34488700 PMC8418963

[16] Ruiz-Medrano J Montero JA Flores-Moreno I Arias L García-Layana A Ruiz-Moreno JM. Myopic maculopathy: current status and proposal for a new classification and grading system (ATN). Prog Retin Eye Res. (2019) 69:5. 10.1016/j.preteyeres.2018.10.005 30391362

[17] Dong L Kang YK Li Y Wei WB Jonas JB. Prevalence and time trends of myopia in children and adolescents in China: a systemic review and meta-analysis. Retina. (2020) 40:399–411. 10.1097/IAE.0000000000002590 31259808

[18] Holden BA Fricke TR Wilson DA Jong M Naidoo KS Sankaridurg P. Global prevalence of myopia and high myopia and temporal trends from 2000 through 2050. Ophthalmology. (2016) 123:1036–42. 10.1016/j.ophtha.2016.01.006 26875007

[19] Wong TY Ferreira A Hughes R Carter G Mitchell P. Epidemiology and disease burden of pathologic myopia and myopic choroidal neovascularization: an evidence-based systematic review. Am J Ophthalmol. (2014) 157:9–25.e12. 10.1016/j.ajo.2013.08.010 24099276

[20] Blencowe H Cousens S Oestergaard MZ Chou D Moller AB Narwal R. National, regional, and worldwide estimates of preterm birth rates in the year 2010 with time trends since 1990 for selected countries: a systematic analysis and implications. Lancet. (2012) 379:2162–72. 10.1016/S0140-6736(12)60820-4 22682464

[21] Stoll BJ Hansen NI Bell EF Walsh MC Carlo WA Shankaran S. Trends in care practices, morbidity, and mortality of extremely preterm neonates, 1993-2012. JAMA. (2015) 314:1039–51. 10.1001/jama.2015.10244 26348753 PMC4787615

[22] Fierson WM. Screening examination of premature infants for retinopathy of prematurity. Pediatrics. (2018) 142:3061. 10.1542/peds.2018-3061 30478242

[23] Jonas JB Aung T Bourne RR Bron AM Ritch R Panda-Jonas S. Glaucoma. Lancet. (2017) 390:2183–93. 10.1016/S0140-6736(17)31469-1 28577860

[24] Collaboration NCDRF. Worldwide trends in diabetes since 1980: a pooled analysis of 751 population-based studies with 4.4 million participants. Lancet. (2016) 387:1513–30. 10.1016/S0140-6736(16)00618-8 27061677 PMC5081106

[25] Wong TY Cheung CMG Larsen M Sharma S Simó R. Diabetic retinopathy. Nat Rev Dis Primers. (2016) 2:16012. 10.1038/nrdp.2016.12 27159554

[26] Li JQ Welchowski T Schmid M Mauschitz MM Holz FG Finger RP. Prevalence and incidence of age-related macular degeneration in Europe: a systematic review and meta-analysis. Br J Ophthalmol. (2020) 104:1077–84. 10.1136/bjophthalmol-2019-314422 31712255

[27] Ting DSW Peng L Varadarajan AV Keane PA Burlina PM Chiang MF. Deep learning in ophthalmology: the technical and clinical considerations. Prog Retin Eye Res. (2019) 72:100759. 10.1016/j.preteyeres.2019.04.003 31048019

[28] Li Y Feng W Zhao X Liu B Zhang Y Chi W. Development and validation of a deep learning system to screen vision-threatening conditions in high myopia using optical coherence tomography images. Br J Ophthalmol. (2020). 10.1136/bjophthalmol-2020-317825 PMC9046742 33355150

[29] Ting DSW Cheung CY-L Lim G Tan GSW Quang ND Gan A. Development and validation of a deep learning system for diabetic retinopathy and related eye diseases using retinal images from multiethnic populations with diabetes. JAMA. (2017) 318:2211–23. 10.1001/jama.2017.18152 29234807 PMC5820739

[30] Son J Shin JY Kim HD Jung K-H Park KH Park SJ. Development and validation of deep learning models for screening multiple abnormal findings in retinal fundus images. Ophthalmology. (2020) 127:85–94. 10.1016/j.ophtha.2019.05.029 31281057

[31] Ung L Jonas JB Lietman TM Chodosh J. COVID-19 and the unfinished agenda of VISION 2020. Am J Ophthalmol. (2020) 224:30–5. 10.1016/j.ajo.2020.11.016 33309690 PMC7831771

[32] Gebrezgabiher G Mekonnen Z Yewhalaw D Hailu A. Reaching the last mile: main challenges relating to and recommendations to accelerate onchocerciasis elimination in Africa. Infect Dis Poverty. (2019) 8:60. 10.1186/s40249-019-0567-z 31269966 PMC6609392

