# Supplementary material for: Prevalence, Years Lived With Disability, and Time Trends for 16 Causes of Blindness and Vision Impairment: Findings Highlight Retinopathy of Prematurity
Source: Front Pediatr. 2022 Mar 11;10:735335. doi: 10.3389/fped.2022.735335 (PMC8962664; doi:10.3389/fped.2022.735335)

**Supplementary Table 1**. List of International Classification of Diseases (ICD) codes mapped to causes of vision loss

| Meningitis | A39-A39.9, A87-A87.9, D86.81, G00-G03.9, G06-G09.9, Z20.811, Z22.31 |
| --- | --- |
| Encephalitis | A83-A85.2, A85.8-A86.0, B94.1, F07.1, G04-G05.8, Z24.1 |
| Onchocerciasis | B73-B73.1 |
| Trachoma | A71-A71.9, A74.0, B94.0 |
| Malaria | B50-B50.0, B50.8-B52.0, B52.8-B53.1, B53.8-B54.0, P37.3-P37.4 |
| Retinopathy of prematurity | P07.2-P07.39, P22-P22.9, P25-P28.9, P61.2, P77-P77.9 |
| Neonatal sepsis and other neonatal infections | A40.1, B95.1, P36-P36.9, P38-P39.9 |
| Hemolytic disease and other neonatal jaundice | P55-P59.9 |
| Neonatal encephalopathy due to birth asphyxia and trauma | P02-P03.9, P10-P15.9, P20-P21.9, P24-P24.9, P52-P52.9, P90-P91.9 |
| Vitamin A deficiency | E50-E50.9, E64.1 |
| Glaucoma | H40-H40.9, H42-H42.8 |
| Cataract | H25-H26.9, H28-H28.8 |
| Age-related macular degeneration | H35.3-H35.389 |
| Diabetic retinopathy | E08-E08.11, E08.3-E08.9, E10-E10.11, E10.3-E11.1, E11.3-E12.1, E12.3-E13.11, E13.3-E14.1, E14.3-E14.9, R73-R73.9, Z13.1, Z83.3 |
| Refractive error | H52-H52.7 |
| Other vision loss | H27-H27.9, H31-H35.23, H35.4-H36.8, H46-H51.9, H53-H54.9 |

**Supplementary Table 2.** Prevalence of cause-specific distance vision loss and blindness in 2019.

|  |  | Moderate vision impairment | | | Severe vision impairment | | | Blindness | | |
| --- | --- | --- | --- | --- | --- | --- | --- | --- | --- | --- |
|  |  | Cases (thousand) | Crude prevalence | Age-standardized prevalence | Number of cases | Crude prevalence | Age-standardized prevalence | Number of cases | Crude prevalence | Age-standardized prevalence |
| **All causes** |  | 253000  [226000-281000] | 3270  [2920-3640] | 3150  [2820-3490] | 33800  [29800-38100] | 437 [385-493] | 420 [371-473] | 41900  [36400-46800] | 542 [470-605] | 523 [457-585] |
| **Communicable diseases** | Meningitis | 146 [110-190] | 1.9 [1.4-2.5] | 1.85 [1.4-2.42] | 44.5 [31.8-61] | 0.6 [0.4-0.8] | 0.6 [0.4-0.8] | 30 [21-42] | 0.4 [0.3-0.5] | 0.4 [0.3-0.5] |
|  | Encephalitis | 160 [128-197] | 2.1 [1.7-2.6] | 1.97 [1.59-2.43] | 39 [29-49] | 0.5 [0.4-0.6] | 0.5 [0.4-0.6] | 28 [21-38] | 0.4 [0.3-0.5] | 0.3 [0.3-0.5] |
|  | Onchocerciasis | 648 [554-751] | 8.4 [7.2-9.7] | 7.7 [6.61-8.93] | 130 [115-146] | 1.7 [1.5-1.9] | 1.6 [1.4-1.8] | 168 [136-201] | 2.2 [1.8-2.6] | 2.0 [1.6-2.4] |
|  | Trachoma | 947 [667-1330] | 12 [9-17] | 11.4 [8.08-16] | 245 [181-321] | 3.2 [2.4-4.1] | 3.0 [2.2-3.9] | 693 [451-1020] | 9.0 [5.8-13.1] | 8.5 [5.5-12.5] |
|  | Malaria | NA | NA | NA | NA | NA | NA | 76 [65-88] | 1.0 [0.84-1.1] | 1.0 [0.8-1.2] |
| **Neonatal disorders** | Retinopathy of prematurity | 2700  [1530-4140] | 35 [20-53] | 35 [19.9-53.6] | 1530  [1080-2040] | 20 [14-26] | 20 [14-27] | 2480 [1890-3080] | 32 [24-40] | 32 [24-39] |
|  | Neonatal sepsis and other neonatal infections | NA | NA | NA | NA | NA | NA | 936 [589-1420] | 12 [8-18] | 12 [7-18] |
|  | Hemolytic disease and other neonatal jaundice | NA | NA | NA | NA | NA | NA | 139 [118-160] | 1.8 [1.5-2.1] | 1.8 [1.5-2.1] |
|  | Neonatal encephalopathy due to birth asphyxia and trauma | NA | NA | NA | NA | NA | NA | 566 [356-816] | 7.3 [4.6-10.5] | 7.3 [4.6-10.5] |
| **Nutritional deficiency** | Vitamin A deficiency | 3470 [2470-4630] | 45 [32-60] | 47 [33-64] | 910 [634-1240] | 12 [8-16] | 12 [9-17] | 425 [253-663] | 5.5 [3.3-8.6] | 5.6 [3.3-8.8] |
| **Eye diseases** | Glaucoma | 3380 [2600-4280] | 44 [34-56] | 42 [33-53] | 613 [450-817] | 7.9 [5.8-10.6] | 7.7 [5.7-10.2] | 3480 [2710-4260] | 45 [35-55] | 45 [35-55] |
|  | Cataract | 67200 [56300-79200] | 869 [727-1020] | 836 [700-979] | 13400 [10900-16400] | 173 [141-212] | 166 [136-204] | 16400 [13900-19300] | 213 [180-249] | 205 [174-239] |
|  | Age-related macular degeneration | 5180 [4110-6440] | 67 [53-83] | 64 [51-79] | 824 [615-1070] | 10 [8-14] | 10 [8-13] | 1790 [1310-2350] | 23 [17-30] | 23 [17-30] |
|  | Diabetic retinopathy | 2640 [1920-3550] | 34 [25-46] | 32 [23-43] | 516 [369-692] | 6.7 [4.8-8.9] | 6.3 [4.5-8.4] | 1040 [739-1470] | 14 [10-19] | 13 [9-18] |
|  | Refractive error | 143000 [126000-161000] | 1850  [1630-2070] | 1780 [1580-1990] | 11000 [9650-12500] | 142 [125-161] | 135 [119-153] | 3600 [3020-4180] | 47 [39-54] | 44 [37-51] |
|  | Other vision loss | 23800 [19900-28100] | 307 [257-364] | 293 [246-345] | 4610  [3710-5610] | 62 [48-73] | 57 [46-69] | 10000  [8470-11700] | 129 [110-151] | 124 [105-144] |

**Supplementary Table 3.** Global prevalence and YLDs with percentage change for moderate and severe vision impairment, 1990 to 2019

|  | Prevalence (per 100,000 population) | | Age-standardized prevalence (per 100,000 population) | | All-age YLDs (per 100,000 population) | | Age-standardized YLD rates (per 100,000 population) | |
| --- | --- | --- | --- | --- | --- | --- | --- | --- |
|  | 2019 counts | Percentage change | 2019 counts | Percentage change | 2019 counts | Percentage change | 2019 counts | Percentage change |
| **Total all causes** |  |  |  |  |  |  |  |  |
| Global | 3710 [330-4130] | 28.88% | 3570 [3200-3960] | 0.95% | 177 [112-269] | 30.37% | 171 [107-259] | -0.20% |
| Central Europe, eastern Europe, and central Asia | 4240 [3740-4770] | 22.21% | 3170 [2830-3520] | -2.65% | 185 [115-284] | 21.48% | 137 [85-209] | -4.44% |
| High income | 2840 [2530-3160] | 21.87% | 2030 [1820-2250] | -1.83% | 141 [88-212] | 25.38% | 94 [60-142] | -3.03% |
| Latin America and Caribbean | 4050 [3610-4480] | 28.51% | 4040 [3610-4470] | -4.51% | 187 [117-282] | 31.70% | 187 [118-283] | -6.67% |
| North Africa and Middle East | 3410 [3060-3780] | 12.82% | 4310 [3850-4780] | -6.13% | 163 [103-248] | 10.86% | 211 [133-321] | -10.85% |
| South Asia | 5230 [4620-5900] | 17.50% | 6440 [5700-7260] | -13.51% | 260 [164-393] | 20.18% | 327 [206-493] | -13.73% |
| Southeast Asia, east Asia, and Oceania | 3700 [3290-4140] | 68.72% | 3260 [2920-3620] | 4.77% | 168 [106-258] | 69.87% | 147 [92-225] | 0.69% |
| Sub-Saharan Africa | 1820 [1630-2030] | -4.47% | 3420 [3030-3840] | -4.58% | 93 [59-140] | -7.86% | 180 [114-271] | -8.11% |
| **Glaucoma** |  |  |  |  |  |  |  |  |
| Global | 52 [39-66] | 67.37% | 50 [38-63] | 8.00% | 2.7 [1.6-4.3] | 59.63% | 2.6 [1.5-4.2] | 3.33% |
| Central Europe, eastern Europe, and central Asia | 50 [38-64] | 40.20% | 32 [25-41] | -3.27% | 2.4 [1.4-3.8] | 35.43% | 1.6 [0.9-2.5] | -6.61% |
| High income | 54 [41-71] | 67.60% | 24 [18-31] | -0.23% | 3.1 [1.8-4.9] | 62.62% | 1.4 [0.8-2.2] | -1.20% |
| Latin America and Caribbean | 81 [62-104] | 85.70% | 85 [65-109] | -4.57% | 4.3 [2.5-6.8] | 78.93% | 4.4 [2.6-7.2] | -8.18% |
| North Africa and Middle East | 50 [38-65] | 31.09% | 83 [62-108] | -9.31% | 2.6 [1.5-4.2] | 23.12% | 4.3 [2.5-6.9] | -14.76% |
| South Asia | 52 [39-68] | 47.23% | 74 [57-95] | -12.81% | 3.1 [1.8-4.9] | 44.48% | 4.4 [2.6-7.0] | -14.96% |
| Southeast Asia, east Asia, and Oceania | 51 [39-65] | 161.63% | 44 [34-56] | 21.23% | 2.3 [1.3-3.7] | 149.43% | 2.0 [1.2-3.2] | 14.92% |
| Sub-Saharan Africa | 35 [27-45] | -7.43% | 100 [77-128] | -4.23% | 1.9 [1.1-3.0] | -11.68% | 5.4 [3.2-8.5] | -8.66% |
| **Cataract** |  |  |  |  |  |  |  |  |
| Global | 1040 [868-1240] | 80.65% | 1000 [836-1180] | 18.59% | 57 [35-87] | 72.46% | 55 [34-83] | 13.88% |
| Central Europe, eastern Europe, and central Asia | 736 [595-885] | 45.20% | 487 [397-583] | 0.22% | 36 [22-56] | 40.09% | 24 [14-37] | -3.15% |
| High income | 748 [611-900] | 61.58% | 347 [284-415] | -1.23% | 43 [26-66] | 56.36% | 20 [12-31] | -2.48% |
| Latin America and Caribbean | 760 [621-909] | 91.31% | 788 [645-944] | -1.03% | 40 [24-62] | 84.51% | 42 [25-64] | -4.77% |
| North Africa and Middle East | 825 [674-983] | 48.06% | 1330 [1090-1580] | 0.49% | 45 [27-69] | 38.48% | 71 [43-109] | -5.93% |
| South Asia | 1570 [1290-1880] | 64.15% | 2150 [1790-2560] | 1.11% | 94 [59-143] | 60.74% | 130 [81-197] | -1.19% |
| Southeast Asia, east Asia, and Oceania | 1240 [1040-1450] | 145.67% | 1080 [915-1260] | 14.46% | 61 [37-93] | 127.68% | 53 [32-80] | 6.23% |
| Sub-Saharan Africa | 448 [375-529] | 11.47% | 1200 [1000-1400] | 11.50% | 25 [16-38] | 6.48% | 67 [41-101] | 6.26% |
| **Age-related macular degeneration** |  |  |  |  |  |  |  |  |
| Global | 78 [61-97] | 66.61% | 74 [59-92] | 10.00% | 3.9 [2.3-6.1] | 58.33% | 3.7 [2.2-5.8] | 4.64% |
| Central Europe, eastern Europe, and central Asia | 53 [42-67] | 47.21% | 34 [27-43] | 4.11% | 2.5 [1.5-3.9] | 42.67% | 1.6 [1.0-2.5] | 0.83% |
| High income | 67 [51-86] | 45.97% | 31 [23-39] | -9.28% | 3.9 [2.3-6.2] | 41.81% | 1.8 [1.1-2.8] | -10.33% |
| Latin America and Caribbean | 55 [43-69] | 92.97% | 56 [44-71] | 1.84% | 2.8 [1.7-4.4] | 86.50% | 2.9 [1.7-4.5] | -1.78% |
| North Africa and Middle East | 76 [58-97] | 40.75% | 120 [92-152] | -0.51% | 4.0 [2.4-6.3] | 32.70% | 6.3 [3.8-9.9] | -6.18% |
| South Asia | 67 [51-85] | 32.75% | 92 [72-115] | -20.52% | 3.9 [2.3-6.1] | 29.27% | 5.4 [3.3-8.4] | -23.11% |
| Southeast Asia, east Asia, and Oceania | 122 [96-153] | 135.77% | 101 [80-125] | 13.86% | 5.3 [3.1-8.3] | 131.27% | 4.4 [2.6-6.9] | 11.04% |
| Sub-Saharan Africa | 40 [31-51] | 1.26% | 108 [85-136] | 5.45% | 2.2 [1.3-3.4] | -4.02% | 5.8 [3.5-9.0] | 0.01% |
| **Refractive error** |  |  |  |  |  |  |  |  |
| Global | 1990 [1760-2240] | 11.96% | 1920 [1700-2150] | -5.39% | 82 [51-126] | 14.16% | 78 [49-121] | -6.32% |
| Central Europe, eastern Europe, and central Asia | 2290 [2030-2590] | 12.80% | 1850 [1640-2070] | -2.89% | 88 [55-138] | 13.16% | 70 [44-109] | -3.70% |
| High income | 1570 [1390-1740] | 5.98% | 1370 [1200-1550] | -2.07% | 67 [42-102] | 9.58% | 54 [34-83] | -3.18% |
| Latin America and Caribbean | 245[1]0 [2150-2740] | 11.74% | 2400 [2120-2680] | -5.86% | 100 [63-153] | 15.55% | 99 [62-150] | -7.31% |
| North Africa and Middle East | 2020 [1790-2280] | 3.77% | 2240 [1990-2510] | -7.35% | 83 [52-128] | 3.59% | 96 [60-148] | -11.18% |
| South Asia | 2920 [2570-3320] | 1.93% | 3380 [2990-3820] | -21.65% | 118 [74-183] | 2.13% | 139 [87-216] | -23.62% |
| Southeast Asia, east Asia, and Oceania | 1790 [1580-2020] | 36.84% | 1600 [1420-1800] | -0.40% | 75 [47-116] | 42.89% | 65 [41-101] | -2.76% |
| Sub-Saharan Africa | 860 [753-978] | 3.01% | 1240 [1090-1400] | 2.30% | 36 [22-55] | 2.12% | 56 [35-85] | 0.68% |
| **Diabetic retinopathy** |  |  |  |  |  |  |  |  |
| Global | 41 [30-55] | 49.88% | 38 [28-52] | 2.54% | 2.2 [1.3-3.5] | 43.46% | 2.1 [1.2-3.3] | -1.06% |
| Central Europe, eastern Europe, and central Asia | 34 [24-46] | 19.49% | 23 [16-30] | -11.02% | 1.7 [1-2.8] | 16.52% | 1.2 [0.6-1.9] | -12.76% |
| High income | 38 [27-52] | 34.38% | 20 [15-28] | -7.08% | 2.3 [1.3-3.6] | 30.22% | 1.2 [0.7-1.9] | -8.84% |
| Latin America and Caribbean | 74 [53-101] | 74.80% | 73 [53-100] | -1.30% | 3.9 [2.2-6.4] | 66.90% | 3.9 [2.2-6.3] | -5.50% |
| North Africa and Middle East | 71 [51-97] | 15.61% | 100 [72-135] | -19.86% | 4.6 [2.6-7.3] | 10.91% | 6.2 [3.5-9.8] | -23.38% |
| South Asia | 24 [17-33] | 29.22% | 31 [22-42] | -16.00% | 1.5 [0.8-2.3] | 25.85% | 1.9 [1.1-3.0] | -17.41% |
| Southeast Asia, east Asia, and Oceania | 53 [38-71] | 108.88% | 42 [31-57] | 4.03% | 2.5 [1.4-4] | 104.92% | 2.0 [1.1-3.2] | 2.25% |
| Sub-Saharan Africa | 15 [10-20] | 8.44% | 35 [25-47] | 9.05% | 0.9 [0.5-1.4] | 3.26% | 2.1 [1.2-3.2] | 3.45% |
| **Other causes of vision loss** |  |  |  |  |  |  |  |  |
| Global | 367 [305-436] | 34.65% | 350 [292-414] | -3.47% | 20 [12-31] | 29.18% | 19 [12-29] | -6.13% |
| Central Europe, eastern Europe, and central Asia | 1020 [838-1220] | 30.11% | 678 [563-813] | -5.68% | 50 [30-76] | 25.02% | 34 [21-52] | -8.87% |
| High income | 313 [255-376] | 33.27% | 191 [157-228] | -2.72% | 18 [11-28] | 29.40% | 11 [7-17] | -5.06% |
| Latin America and Caribbean | 510 [419-611] | 56.92% | 514 [423-615] | -6.56% | 26 [16-41] | 48.39% | 27 [16-41] | -10.33% |
| North Africa and Middle East | 240 [195-292] | 10.86% | 314 [256-383] | -6.06% | 14 [9-22] | -0.52% | 18 [11-28] | -13.57% |
| South Asia | 397 [327-482] | 31.01% | 507 [420-608] | -5.36% | 24 [15-37] | 26.17% | 31 [19-47] | -7.70% |
| Southeast Asia, east Asia, and Oceania | 357 [303-422] | 83.58% | 296 [253-344] | -2.03% | 17 [11-26] | 74.55% | 14 [9-22] | -7.54% |
| Sub-Saharan Africa | 132 [109-160] | 23.70% | 278 [228-335] | 25.87% | 8 [5-12] | 19.27% | 16 [10-24] | 21.11% |
| **Vitamin A deficiency** |  |  |  |  |  |  |  |  |
| Global | 57 [40-76] | 7.78% | 60 [42-80] | 23.12% | 3.5 [2.0-5.7] | 6.36% | 3.7 [2.1-6.0] | 20.73% |
| Central Europe, eastern Europe, and central Asia | 12 [8-16] | 35.54% | 15 [10-21] | 58.83% | 0.7 [0.4-1.1] | 32.09% | 0.9 [0.5-1.5] | 54.57% |
| High income | 0 [0-0] | NA | 0 [0-0] | NA | 0 [0-0] | NA | 0 [0-0] | NA |
| Latin America and Caribbean | 64 [45-87] | -9.07% | 68 [47-92] | 10.3% | 3.8 [2.1-6.3] | -14.95% | 4.0 [2.3-6.6] | 4.89% |
| North Africa and Middle East | 43 [29-59] | 2.71% | 42 [29-58] | 25.25% | 2.6 [1.5-4.4] | -7.29% | 2.6 [1.4-4.3] | 11.25% |
| South Asia | 107 [76-143] | -9.02% | 110 [78-146] | 4.07% | 7.0 [4.0-11.3] | -9.04% | 7.1 [4.1-11.3] | 0.70% |
| Southeast Asia, east Asia, and Oceania | 43 [31-57] | 4.79% | 53 [37-72] | 33.05% | 2.2 [1.3-3.6] | 1.84% | 2.7 [1.5-4.5] | 28.66% |
| Sub-Saharan Africa | 77 [53-106] | 4.12% | 69 [49-91] | 12.39% | 5.2 [3.0-8.5] | -0.32% | 4.5 [2.7-7.3] | 7.07% |
| **Maternal and** **neonatal disorders** |  |  |  |  |  |  |  |  |
| Global | 55 [34-80] | 13.73% | 55 [34-80] | 14.78% | 4.6 [2.6-7.5] | 11.07% | 4.7 [2.6-7.6] | 12.27% |
| Central Europe, eastern Europe, and central Asia | 42 [23-64] | 20.61% | 42 [23-63] | 20.13% | 3.5 [1.7-5.9] | 14.49% | 3.5 [1.7-5.9] | 14.48% |
| High income | 51 [29-79] | 15.13% | 52 [29-79] | 15.65% | 4.0 [1.9-7.1] | 10.18% | 4.1 [2.0-7.1] | 10.60% |
| Latin America and Caribbean | 53 [30-79] | 20.16% | 53 [30-79] | 19.80% | 4.4 [2.1-7.4] | 14.69% | 4.4 [2.1-7.4] | 14.60% |
| North Africa and Middle East | 72 [44-105] | 30.60% | 72 [44-105] | 30.26% | 6.1 [3.5-10] | 27.58% | 6.1 [3.5-9.9] | 27.37% |
| South Asia | 68 [42-99] | -1.10% | 68 [43-99] | -1.00% | 6.2 [3.5-10] | -1.11% | 6.2 [3.5-10] | -0.87% |
| Southeast Asia, east Asia, and Oceania | 39 [24-57] | 8.05% | 39 [24-57] | 8.38% | 3.5 [2.0-5.6] | 7.50% | 3.5 [2.0-5.6] | 8.05% |
| Sub-Saharan Africa | 64 [37-95] | 6.13% | 64 [37-96] | 6.41% | 4.8 [2.5-8.2] | 3.11% | 4.8 [2.4-8.2] | 2.95% |
| **Communicable diseases** |  |  |  |  |  |  |  |  |
| Global | 31 [24-39] | -35.80% | 29 [22-37] | -52.40% | 1.8 [1.1-2.8] | -36.20% | 1.7 [1-2.7] | -51.94% |
| Central Europe, eastern Europe, and central Asia | 3.5 [2.7-4.5] | -22.91% | 3.0 [2.2-3.9] | -31.53% | 0.2 [0.1-0.3] | -28.93% | 0.2 [0.1-0.3] | -34.89% |
| High income | 2.0 [1.6-2.6] | -39.27% | 1.7 [1.3-2.2] | -46.91% | 0.1 [0.1-0.2] | -39.96% | 0.1 [0.1-0.2] | -48.10% |
| Latin America and Caribbean | 5.7 [4.1-7.5] | -44.83% | 5.5 [4.0-7.3] | -59.71% | 0.3 [0.2-0.5] | -49.94% | 0.3 [0.2-0.5] | -62.46% |
| North Africa and Middle East | 7.6 [5.5-10.5] | -83.50% | 9.5 [6.7-13.3] | -89.51% | 0.5 [0.3-0.8] | -83.20% | 0.6 [0.3-0.9] | -89.35% |
| South Asia | 26 [17-39] | -28.57% | 33 [22-49] | -49.24% | 1.6 [0.9-2.7] | -31.05% | 2.0 [1.1-3.4] | -50.09% |
| Southeast Asia, east Asia, and Oceania | 6.1 [4.3-8.4] | -53.97% | 5.1 [3.7-7.0] | -72.70% | 0.3 [0.2-0.5] | -55.57% | 0.2 [0.1-0.4] | -73.55% |
| Sub-Saharan Africa | 152 [121-188] | -55.17% | 333 [265-414] | -53.84% | 9.1 [5.6-14.0] | -55.41% | 19 [12-30] | -54.28% |

Data was expressed as estimate [95% uncertainty interval]

**Supplementary Table 4.** Global prevalence and YLDs with percentage change for blindness, 1990 to 2019

|  | Prevalence (per 100,000 population) | | Age-standardized prevalence (per 100,000 population) | | All-age YLDs (per 100,000 population) | | Age-standardized YLD rates (per 100,000 population) | |
| --- | --- | --- | --- | --- | --- | --- | --- | --- |
|  | 2019 counts | Percentage change | 2019 counts | Percentage change | 2019 counts | Percentage change | 2019 counts | Percentage change |
| **Total all causes** |  |  |  |  |  |  |  |  |
| Global | 542 [470-605] | 0.86% | 523 [457-585] | -27.14% | 98 [65-140] | 1.07% | 95 [63-135] | -26.72% |
| Central Europe, eastern Europe, and central Asia | 332 [287-375] | 3.05% | 239 [208-268] | -23.50% | 60 [40-86] | 3.03% | 44 [29-62] | -23.17% |
| High income | 275 [238-311] | 14.20% | 154 [134-173] | -21.68% | 50 [33-71] | 13.76% | 28 [19-40] | -21.59% |
| Latin America and Caribbean | 602 [525-674] | 14.91% | 610 [531-684] | -32.67% | 110 [73-157] | 14.94% | 111 [74-158] | -32.26% |
| North Africa and Middle East | 476 [408-542] | -18.80% | 699 [592-801] | -41.54% | 87 [57-124] | -18.45% | 127 [84-183] | -41.10% |
| South Asia | 652 [567-732] | -17.69% | 895 [780-1010] | -46.69% | 117 [78-165] | -17.46% | 160 [107-226] | -46.27% |
| Southeast Asia, east Asia, and Oceania | 670 [585-757] | 21.04% | 586 [516-658] | -32.43% | 122 [82-174] | 21.22% | 107 [71-151] | -31.81% |
| Sub-Saharan Africa | 453 [399-507] | -26.50% | 990 [852-1120] | -27.50% | 83 [56-117] | -21.18% | 179 [120-255] | -27.21% |
| **Glaucoma** |  |  |  |  |  |  |  |  |
| Global | 45 [35-55] | 7.77% | 45 [35-54] | -31.94% | 8.1 [5.1-12.0] | 7.98% | 8.0 [5.0-11.9] | -31.66% |
| Central Europe, eastern Europe, and central Asia | 41 [32-51] | 2.14% | 27 [21-33] | -32.06% | 7.5 [4.7-11.1] | 2.13% | 4.9 [3.1-7.3] | -31.84% |
| High income | 71 [57-88] | 32.40% | 31 [25-38] | -23.23% | 13.0 [8.3-19.1] | 32.06% | 5.6 [3.6-8.3] | -23.16% |
| Latin America and Caribbean | 55 [42-67] | 30.73% | 58 [44-71] | -36.10% | 9.8 [6.2-14.8] | 31.08% | 10.4 [6.5-15.6] | -35.71% |
| North Africa and Middle East | 71 [54-88] | -13.36% | 126 [97-157] | -40.28% | 12.7 [7.9-19.1] | -12.97% | 22.6 [14.0-33.8] | -39.89% |
| South Asia | 32 [24-40] | 6.05% | 50 [38-62] | -38.66% | 5.7 [3.5-8.6] | 6.55% | 8.7 [5.4-13.0] | -38.08% |
| Southeast Asia, east Asia, and Oceania | 33 [25-42] | 13.27% | 29 [23-37] | -48.43% | 6.0 [3.6-9.0] | 13.78% | 5.3 [3.3-7.9] | -47.98% |
| Sub-Saharan Africa | 45 [36-56] | -25.68% | 145 [114-177] | -23.63% | 8.2 [5.1-12.2] | -25.42% | 25.9 [16.5-38.6] | -23.26% |
| **Cataract** |  |  |  |  |  |  |  |  |
| Global | 213 [180-249] | -1.11% | 205 [174-239] | -32.75% | 38 [26-56] | -0.96% | 37 [25-54] | -32.48% |
| Central Europe, eastern Europe, and central Asia | 68 [54.83] | -6.65 | 46 [37-56] | -34.94% | 12 [8-18] | -6.63% | 8.3 [5.4-12.1] | -34.70% |
| High income | 46 [38-57] | 20.05% | 23 [19-28] | -23.30% | 8.4 [5.4-12.5] | 19.50% | 4.3 [2.7-6.4] | -23.29% |
| Latin America and Caribbean | 180 [149-216] | 8.65% | 187 [155-224] | -43.76% | 33 [21-48] | 8.75% | 34 [22-50] | -43.47% |
| North Africa and Middle East | 128 [102-157] | -33.01% | 209 [167-258] | -54.28% | 23 [15-35] | -32.70% | 38 [25-57] | -53.97% |
| South Asia | 348 [295-406] | -13.95% | 511 [436-594] | -47.27% | 62 [42-89] | -13.84% | 91 [61-131] | -47.00% |
| Southeast Asia, east Asia, and Oceania | 278 [235-329] | 15.92% | 243 [207-285] | -42.87% | 50 [34-73] | 16.09% | 44 [30-64] | -42.46% |
| Sub-Saharan Africa | 143 [121-165] | -31.46% | 366 [309-431] | -31.06% | 26 [17-37] | -31.27% | 66 [44-96] | -30.85% |
| **Age-related macular degeneration** |  |  |  |  |  |  |  |  |
| Global | 23 [17-30] | 13.50% | 23 [17-30] | -27.79% | 4.2 [2.6-6.3] | 13.67% | 4.1 [2.6-6.2] | -27.56% |
| Central Europe, eastern Europe, and central Asia | 15 [10-20] | 19.10% | 9.6 [6.7-12.9] | -16.86% | 2.7 [1.6-4.1] | 19.01% | 1.7 [1.1-2.7] | -16.71% |
| High income | 54 [42-70] | 22.54% | 24 [18-31] | -28.51% | 9.9 [6.3-15.0] | 22.20% | 4.3 [2.8-6.5] | -28.44% |
| Latin America and Caribbean | 12 [8-16] | 53.26% | 12 [8-16] | -21.47% | 2.21 [1.3-3.2] | 53.67% | 2.2 [1.3-3.4] | -21.01% |
| North Africa and Middle East | 30 [21-40] | 7.55% | 49 [34-65] | -23.77% | 5.4 [3.2-8.2] | 8.03% | 8.8 [5.2-13.4] | -23.29% |
| South Asia | 16 [11-23] | -8.24% | 23 [16-32] | -41.14% | 2.9 [1.7-4.5] | -7.82% | 4.1 [2.4-6.3] | -40.68% |
| Southeast Asia, east Asia, and Oceania | 22 [15-30] | 52.60% | 18 [13-25] | -26.84% | 3.9 [2.3-6.2] | 53.33% | 3.3 [2.0-5.1] | -26.24% |
| Sub-Saharan Africa | 12 [8-16] | -21.81% | 33 [23-45] | -18.86% | 2.1 [1.3-3.2] | -21.53% | 5.9 [3.5-9.0] | -18.47% |
| **Refractive error** |  |  |  |  |  |  |  |  |
| Global | 47 [39-54] | -5.83% | 44 [37-51] | -25.35% | 8.5 [5.6-13.3] | -5.53% | 8.1 [5.3-11.7] | -24.86% |
| Central Europe, eastern Europe, and central Asia | 6.9 [5.5-8.5] | 2.89% | 5.8 [4.6-7.2] | -8.39% | 1.3 [0.8-1.9] | 2.94% | 1.1 [0.7-1.6] | -8.09% |
| High income | 7.3 [5.8-9.0] | -10.53% | 5.9 [4.5-7.5] | -20.93% | 1.4 [0.9-2.0] | -10.60% | 1.1 [0.7-1.7] | -20.74% |
| Latin America and Caribbean | 36 [30-43] | 1.50% | 35 [29-42] | -29.00% | 6.6 [4.3-9.7] | 1.53% | 6.5 [4.3-9.5] | -28.67% |
| North Africa and Middle East | 30 [24-36] | -19.00% | 35 [29-41] | -32.45% | 5.5 [3.6-8.2] | -18.86% | 6.4 [4.3-9.4] | -32.12% |
| South Asia | 82 [69-96] | -26.32% | 100 [83-117] | -48.90% | 15 [21-10] | -26.00% | 18 [12-26] | -48.41% |
| Southeast Asia, east Asia, and Oceania | 63 [52-74] | 16.65% | 53 [44-62] | -22.92% | 12 [8-17] | 16.68% | 9.8 [6.5-14.3] | -22.47% |
| Sub-Saharan Africa | 23 [19-27] | -11.17% | 38 [31-45] | -13.47% | 4.2 [2.7-6.2] | -10.79% | 6.9 [4.5-10.1] | -13.07% |
| **Diabetic retinopathy** |  |  |  |  |  |  |  |  |
| Global | 13 [10-19] | 54.74% | 13 [9-18] | 10.25% | 2.5 [1.4-3.9] | 22.65% | 2.3 [1.3-3.6] | 10.31% |
| Central Europe, eastern Europe, and central Asia | 3.3 [2.2-4.7] | 22.93% | 2.2 [1.5-3.2] | -6.64% | 0.6 [0.3-1.0] | 54.60% | 0.4 [0.2-0.7] | -6.72% |
| High income | 15 [11-21] | 18.85% | 8.6 [6.2-12.0] | -13.94% | 2.7 [1.6-4.2] | 18.53% | 1.6 [0.9-2.5] | -13.95% |
| Latin America and Caribbean | 52 [31-57] | 33.86% | 41 [30-56] | -20.31% | 7.7 [11.7-4.5] | 33.92% | 7.5 [4.4-11.4] | -20.11% |
| North Africa and Middle East | 11 [8-16] | 42.22% | 15 [11-23] | -0.45% | 2.1 [1.2-3.4] | 42.97% | 2.8 [1.6-4.5] | 0.16% |
| South Asia | 11 [7-15] | 45.64% | 13 [9-19] | 6.66% | 1.9 [1.1-3.0] | 45.40% | 2.4 [1.4-3.7] | 6.81% |
| Southeast Asia, east Asia, and Oceania | 15 [10-21] | 164.91% | 11 [16-8] | 43.80% | 2.7 [1.5-4.3] | 165.07% | 2.1 [1.2-3.3] | 44.43% |
| Sub-Saharan Africa | 4.4 [3.1-6.3] | 19.58% | 9.3 [6.6-13.1] | 20.62% | 0.8 [0.5-1.3] | 19.62% | 1.7 [1.0-2.7] | 20.63% |
| **Other causes of vision loss** |  |  |  |  |  |  |  |  |
| Global | 129 [110-151] | 6.15% | 124 [105-144] | -20.51% | 24 [16-34] | 6.01% | 23 [15-33] | -20.33% |
| Central Europe, eastern Europe, and central Asia | 162 [135-188] | 3.82% | 114 [96-132] | -24.46% | 29 [19-43] | 3.69% | 21 [14-30] | -24.26% |
| High income | 50 [41-60] | -5.23% | 32 [26-39] | -28.58% | 9.2 [5.9-13.5] | -5.61% | 5.9 [3.7-8.7] | -28.60% |
| Latin America and Caribbean | 219 [183-256] | 14.31% | 221 [184-259] | -30.17% | 40 [26-58] | 14.16% | 40 [27-58] | -29.88% |
| North Africa and Middle East | 148 [122-179] | -11.73% | 206 [170-248] | -31.48% | 27 [17-39] | -11.61% | 38 [24-55] | -31.22% |
| South Asia | 78 [65-90] | -6.09% | 103 [86-119] | -26.98% | 14 [9-20] | -6.37% | 18 [13-27] | -26.84% |
| Southeast Asia, east Asia, and Oceania | 185 [157-215] | 25.93% | 159 [184-135] | -24.62% | 34 [22-49] | 25.77% | 29 [19-42] | -24.19% |
| Sub-Saharan Africa | 114 [97-133] | -16.35% | 244 [287-204] | -11.72% | 21 [14-30] | -16.18% | 44 [29-64] | -11.46% |
| **Vitamin A deficiency** |  |  |  |  |  |  |  |  |
| Global | 5.5 [3.3-8.6] | -32.32% | 5.6 [3.3-8.8] | -28.74% | 1.0 [0.5-1.7] | -32.12% | 1 [0.5-1.8] | -28.50% |
| Central Europe, eastern Europe, and central Asia | 0.4 [0.1-0.7] | 8.90% | 0.4 [0.1-0.7] | 11.69% | 0.1 [0-0.1] | 8.82% | 0.1 [0-0.1] | 11.62% |
| High income | 0 [0-0] | NA | 0 [0-0] | NA | 0 [0-0] | NA | 0 [0-0] | NA |
| Latin America and Caribbean | 2.5 [1.3-4.0] | -45.06% | 2.4 [1.2-4.0] | -48.16% | 0.5 [0.2-0.8] | -45.05% | 0.5 [0.2-0.8] | -48.13% |
| North Africa and Middle East | 1.4 [0.6-2.7] | -37.87% | 1.5 [0.6-2.7] | -38.10% | 0.3 [0.1-0.5] | -37.73% | 0.3 [0.1-0.5] | -37.87% |
| South Asia | 5.9 [3.4-9.3] | -46.25% | 6.1 [3.5-9.1] | -47.20% | 1.1 [0.6-1.9] | -45.83% | 1.1 [0.6-1.9] | -46.66% |
| Southeast Asia, east Asia, and Oceania | 7.5 [4.5-11.6] | -34.09% | 8.0 [4.6-12.7] | -27.22% | 1.4 [0.7-2.4] | -34.01% | 1.5 [0.8-2.6] | -26.99% |
| Sub-Saharan Africa | 12 [7-19] | -35.46% | 11 [7-17] | -32.38% | 2.2 [1.1-3.9] | -35.28% | 2.1 [1.1-3.5] | -32.16% |
| **Maternal and neonatal disorders** |  |  |  |  |  |  |  |  |
| Global | 53 [44-63] | 33.53% | 52 [43-62] | 32.88% | 9.9 [6.4-14.5] | 33.96% | 9.8 [6.3-14.3] | 33.38% |
| Central Europe, eastern Europe, and central Asia | 36 [30-43] | 15.59% | 34 [28-41] | 12.02% | 6.7 [4.3-9.6] | 15.62% | 6.4 [4-9.1] | 12.06% |
| High income | 30 [26-36] | 1.52% | 29 [24-35] | -1.88% | 5.7 [3.6-8.2] | 1.54% | 5.4 [3.4-7.8] | -1.84% |
| Latin America and Caribbean | 54 [45-66] | 29.74% | 53 [44-64] | 28.16% | 10 [7-15] | 29.96% | 9.9 [6.3-14.5] | 28.51% |
| North Africa and Middle East | 53 [44-62] | 3.07% | 51 [43-61] | 1.32% | 10 [6-14] | 3.30% | 9.6 [6.3-13.8] | 1.68% |
| South Asia | 55 [45-67] | 16.75% | 55 [45-67] | 17.66% | 10.3 [6.5-15.3] | 17.58% | 10 [7-15] | 18.82% |
| Southeast Asia, east Asia, and Oceania | 63 [49-77] | 63.81% | 60 [47-72] | 58.06% | 12 [8-18] | 64.19% | 11 [7-16] | 58.71% |
| Sub-Saharan Africa | 60 [46-74] | 34.19% | 60 [46-75] | 37.57% | 11 [7-17] | 35.14% | 11 [7-17] | 38.66% |
| **Communicable diseases** |  |  |  |  |  |  |  |  |
| Global | 13 [9-18] | -59.75% | 12 [9-17] | -71.27% | 2.3 [1.4-3.8] | -59.56% | 2.2 [1.3-3.6] | -71.06% |
| Central Europe, eastern Europe, and central Asia | 0.2 [0.1-0.3] | -49.94% | 0.2 [0.1-0.2] | -56.85% | 0 [0-0.1] | -49.96% | 0 [0-0] | -56.85% |
| High income | 0.1 [0.1-0.1] | -65.25% | 0.1 [0-0.1] | -73.78% | 0 [0-0] | -65.27% | 0 [0-0] | -73.79% |
| Latin America and Caribbean | 0.8 [0.5-1.1] | -73.84% | 0.8 [0.5-1.1] | -83.38% | 0.1 [0.1-0.2] | -73.67% | 0.1 [0.1-0.2] | -83.20% |
| North Africa and Middle East | 4.7 [2.9-7.1] | -77.12% | 6.8 [4.2-10.5] | -84.50% | 0.9 [0.4-1.5] | -76.94% | 1.2 [0.6-2.1] | -84.37% |
| South Asia | 24 [15-36] | -69.78% | 35 [22-51] | -81.15% | 4.3 [2.4-7.4] | -69.75% | 6.1 [3.4-10.2] | -81.04% |
| Southeast Asia, east Asia, and Oceania | 4.3 [2.7-6.6] | -69.41% | 3.6 [2.3-5.5] | -83.97% | 0.8 [0.4-1.4] | -69.17% | 0.7 [0.4-1.1] | -83.72% |
| Sub-Saharan Africa | 40 [30-52] | -61.19% | 83 [62-109] | -61.39% | 7.3 [4.5-11.4] | -61.06% | 15 [9-24] | -61.29% |

Data was expressed as estimate [95% uncertainty interval]

**Supplementary Table 5.** Prevalence changes of cause-specific distance vision loss and blindness.1990-2019.

|  |  | Moderate vision impairment | | | Severe vision impairment | | | Blindness | | |
| --- | --- | --- | --- | --- | --- | --- | --- | --- | --- | --- |
|  |  | Cases | Crude prevalence | Age-standardized prevalence | Number of cases | Crude prevalence | Age-standardized prevalence | Number of cases | Crude prevalence | Age-standardized prevalence |
| **All causes** |  | 85.63% | 28.35% | 1.48% | 92.39% | 33.02% | -2.85% | 45.88% | 0.86% | -27.14% |
| **Communicable diseases** | Meningitis | -16.79% | -42.47% | -47.47% | -22.17% | -46.19% | -48.07% | -53.45% | -67.82% | -70.14% |
|  | Encephalitis | 30.60% | -9.70% | -24.38% | 12.33% | -22.33% | -31.78% | -36.81% | -56.31% | -62.79% |
|  | Onchocerciasis | 3.61% | -28.36% | -47.42% | 0.78% | -30.32% | -46.65% | -34.91% | -55.00% | -67.08% |
|  | Trachoma | -15.16% | -41.34% | -58.57% | -12.66% | -39.61% | -56.68% | -46.17% | -62.78% | -74.45% |
|  | Malaria | NA | NA | NA | NA | NA | NA | 38.47% | -4.26% | 0.59% |
| **Neonatal disorders** | Retinopathy of prematurity | 68.10% | 16.23% | 17.23% | 58.48% | 9.58% | 10.71% | 40.47% | -2.87% | -3.23% |
|  | Neonatal sepsis and other neonatal infections | NA | NA | NA | NA | NA | NA | 573.33% | 365.55% | 367.44% |
|  | Hemolytic disease and other neonatal jaundice | NA | NA | NA | NA | NA | NA | 53.72% | 6.28% | 7.40% |
|  | Neonatal encephalopathy due to birth asphyxia and trauma | NA | NA | NA | NA | NA | NA | 306.74% | 181.23% | 177.55% |
| **Nutritional deficiency** | Vitamin A deficiency | 57.11% | 8.63% | 24.53% | 51.39% | 4.68% | 17.98% | -2.11% | -32.32% | -28.74% |
| **Eye diseases** | Glaucoma | 87.46% | 29.61% | 1.88% | 99.42% | 37.88% | -2.51% | 47.29% | 1.84% | -28.61% |
|  | Cataract | 147.46% | 71.10% | 10.32% | 116.08% | 49.40% | -3.19% | 55.86% | 7.77% | -31.94% |
|  | Age-related macular degeneration | 167.27% | 84.79% | 21.06% | 134.83% | 62.37% | 7.55% | 43.03% | -1.11% | -32.75% |
|  | Diabetic retinopathy | 146.42% | 70.38% | 12.49% | 111.53% | 46.25% | -3.40% | 64.16% | 13.50% | -27.79% |
|  | Refractive error | 121.29% | 53.00% | 4.36% | 96.23% | 35.68% | -5.88% | 123.81% | 54.74% | 10.25% |
|  | Other vision loss | 61.03% | 11.34% | -5.04% | 74.66% | 20.77% | -9.80% | 36.20% | -5.83% | -25.35% |

**Supplementary Figure 1**. Age-standardized YLD rates of blindness and distance vision impairment by location in 2019

**
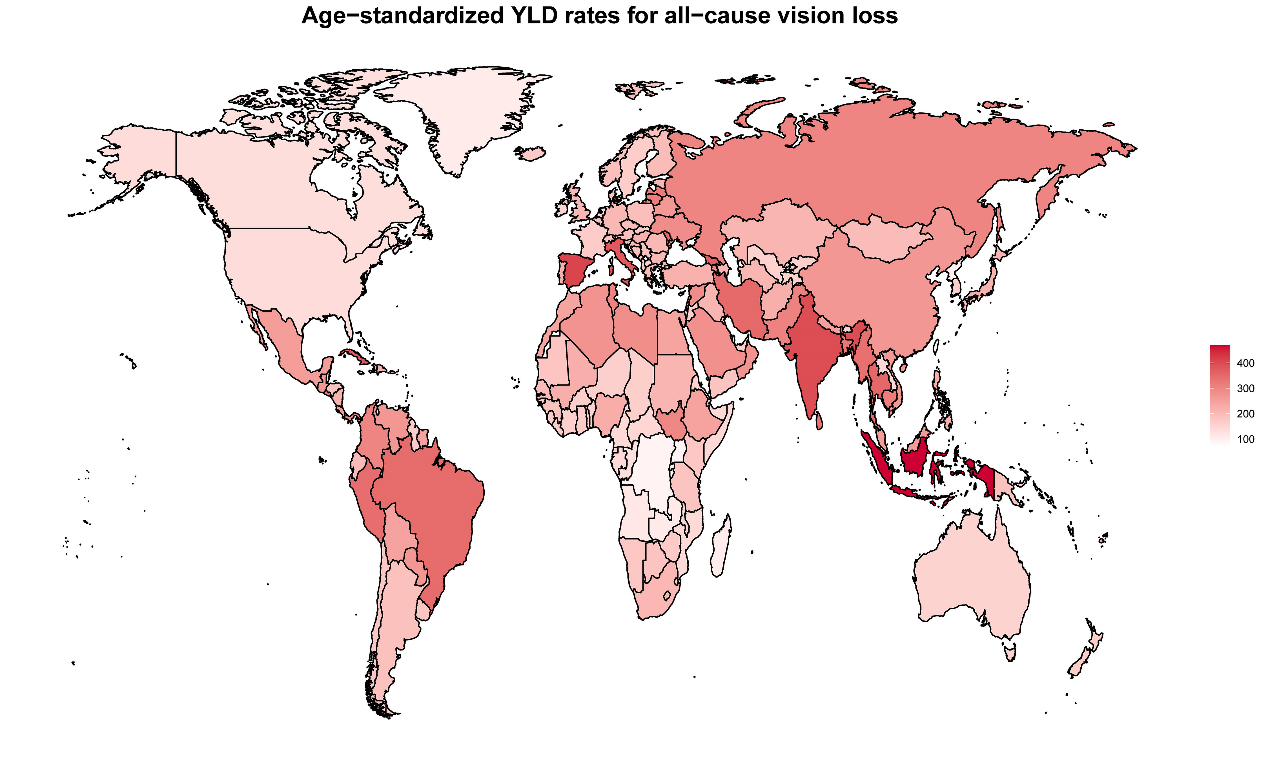
**

**Supplementary Figure 2**. The predominate cause for age-standardized YLD of blindness and distance vision impairment in both sexes combined, 2019


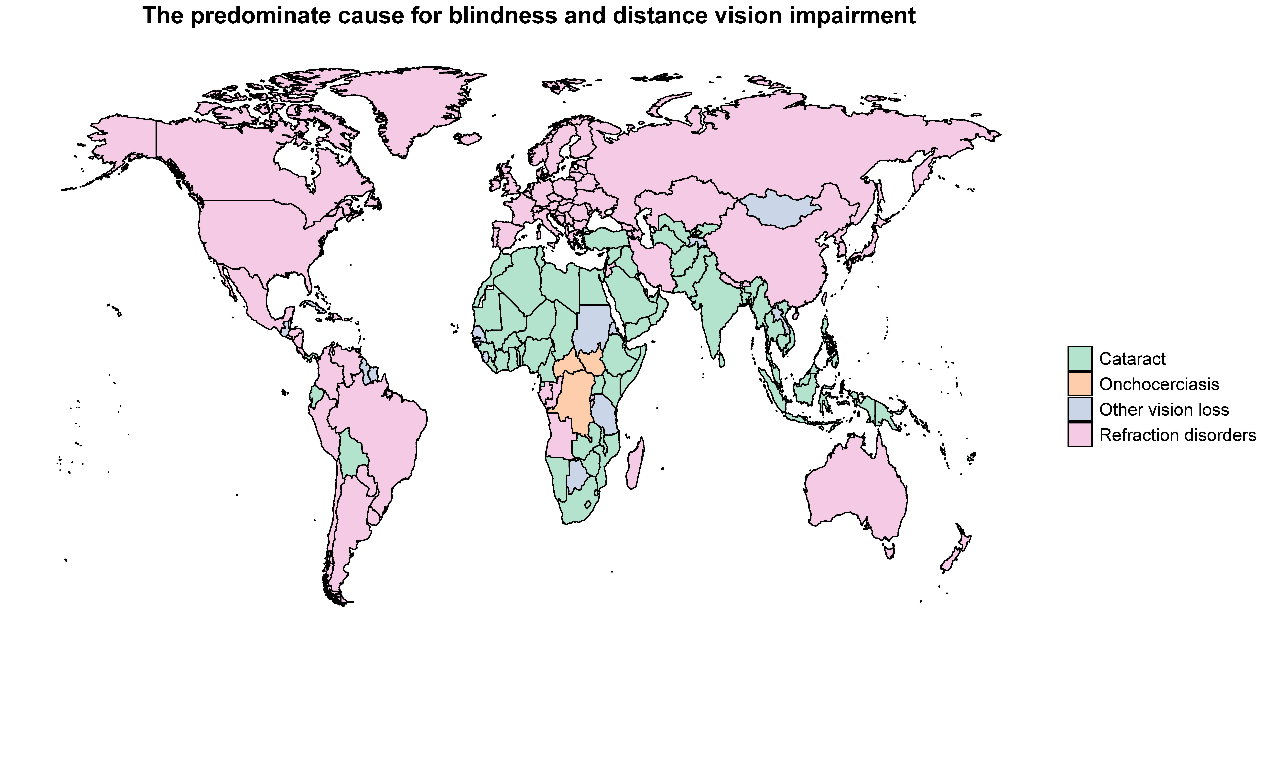


**Supplementary Figure 3**. The age-standardized YLD of blindness and distance vision impairment due to age-related macular degeneration in both sexes combined, 2019


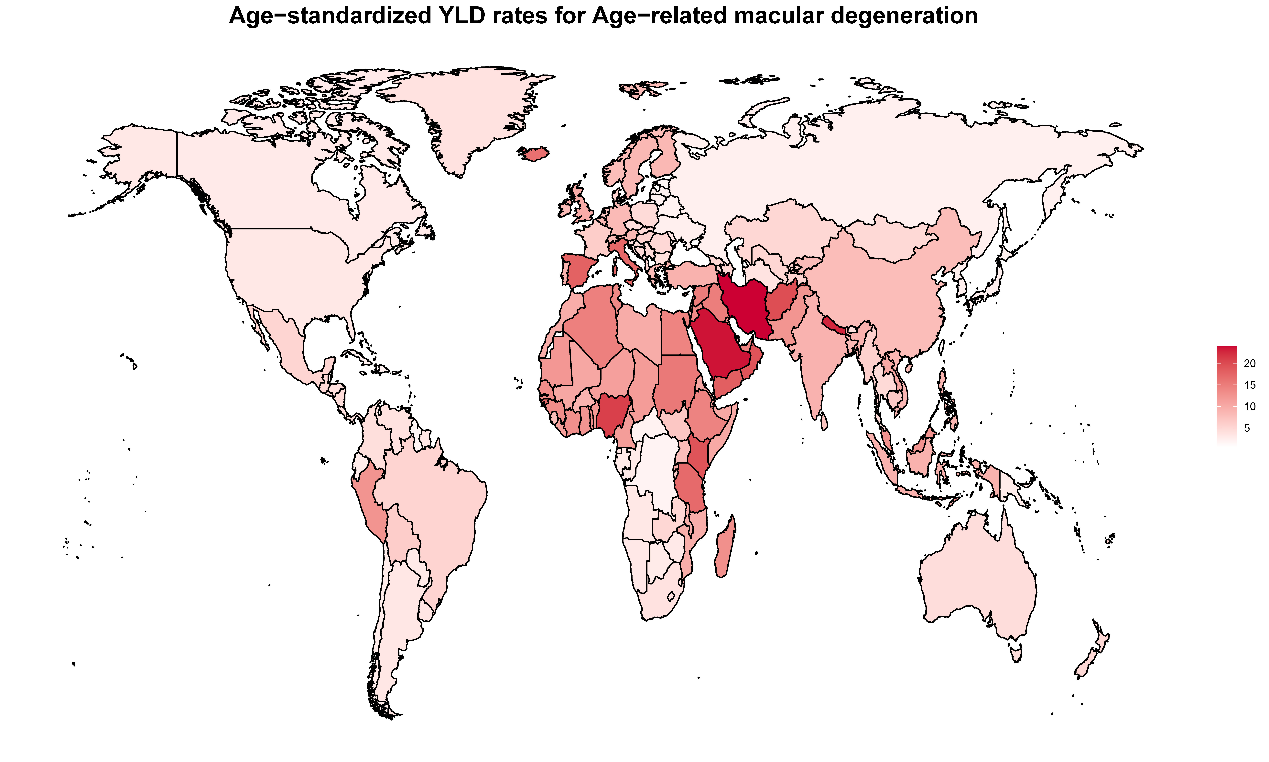


**Supplementary Figure 4**. The age-standardized YLD of blindness and distance vision impairment due to cataract in both sexes combined, 2019


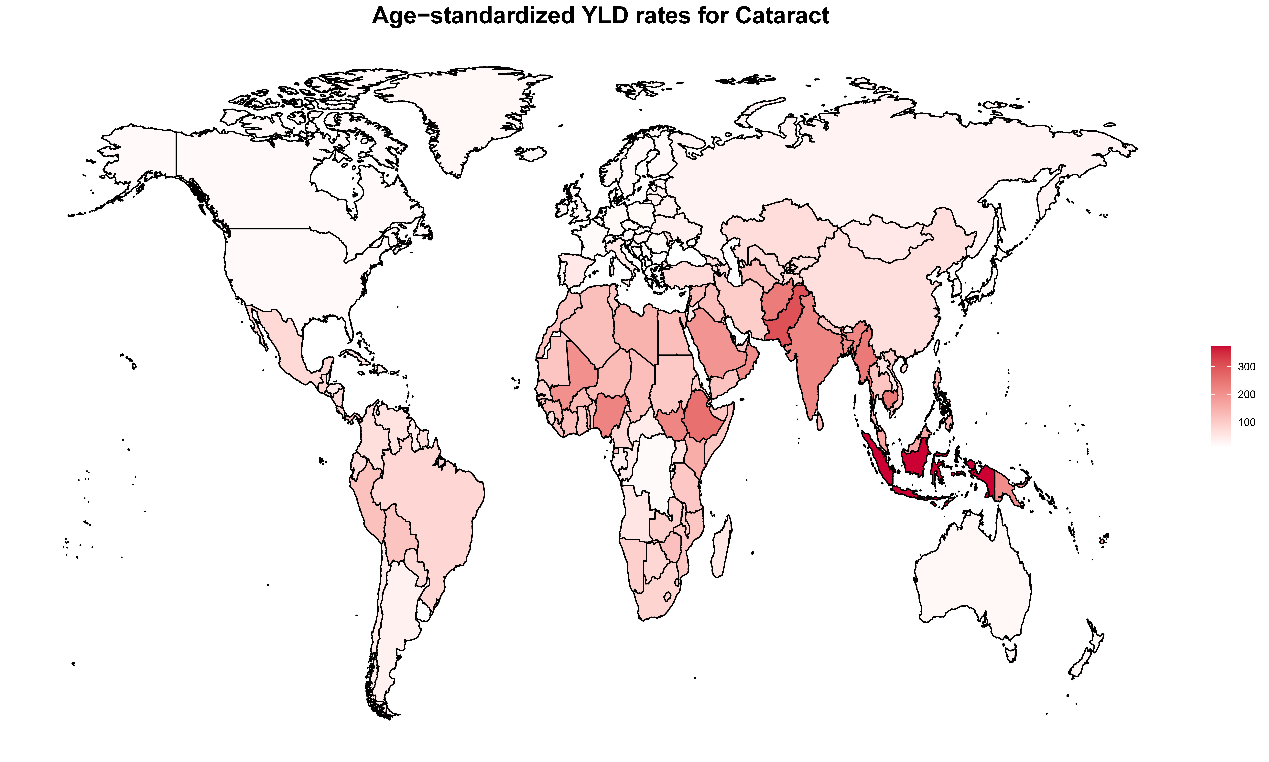


**Supplementary Figure 5**. The age-standardized YLD of blindness and distance vision impairment due to diabetic retinopathy in both sexes combined, 2019


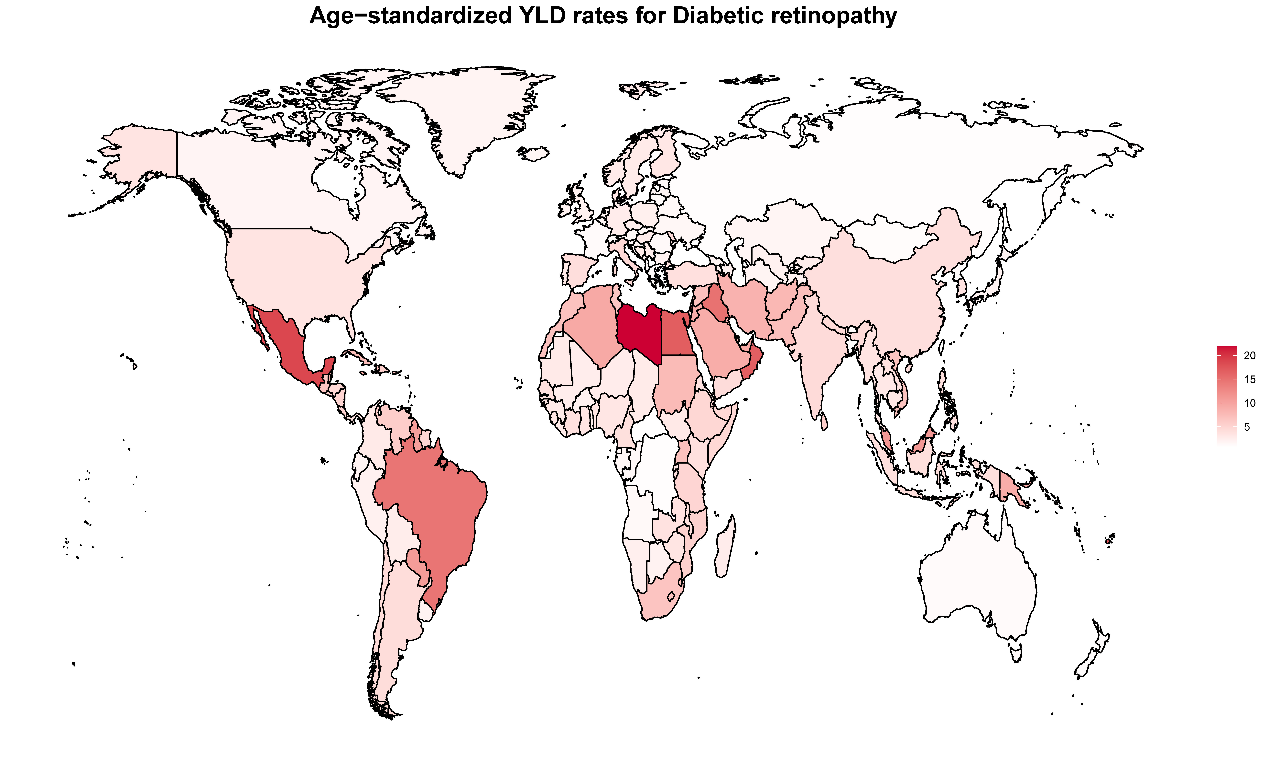


**Supplementary Figure 6**. The age-standardized YLD of blindness and distance vision impairment due to encephalitis in both sexes combined, 2019


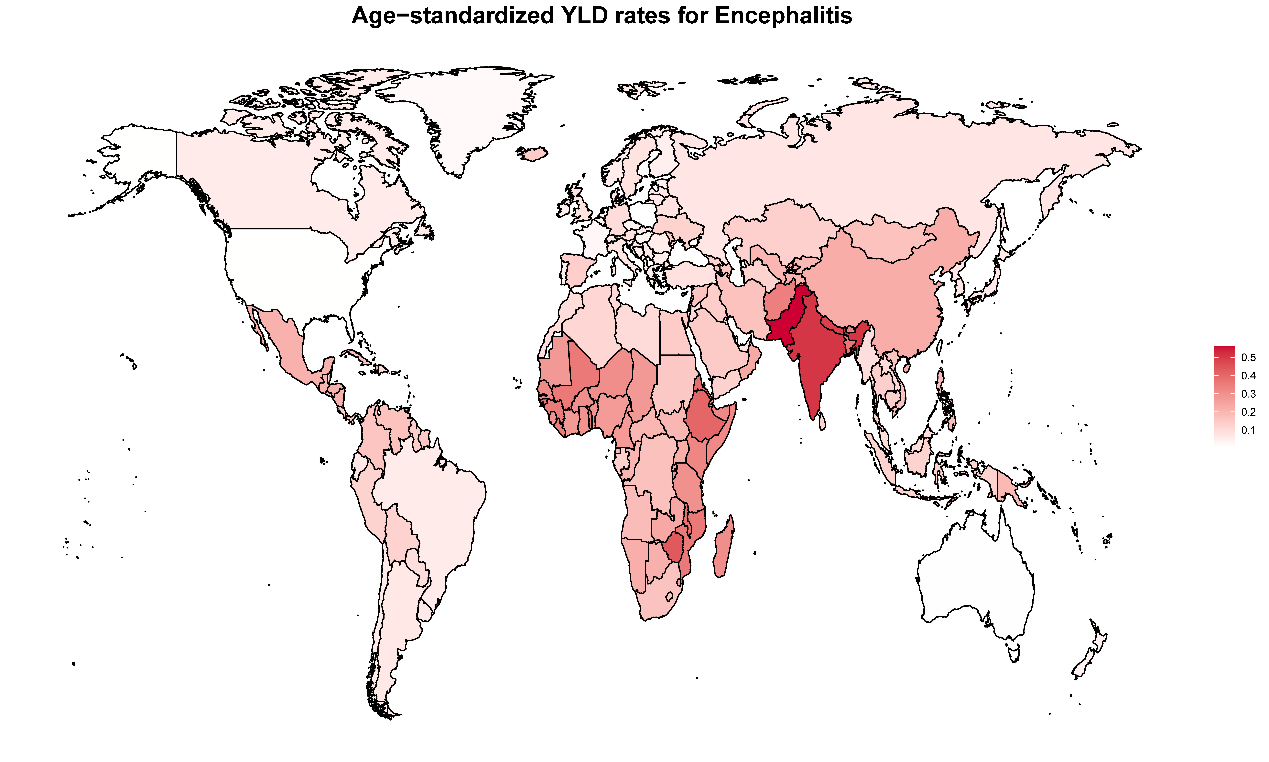


**Supplementary Figure 7**. The age-standardized YLD of blindness and distance vision impairment due to glaucoma in both sexes combined, 2019


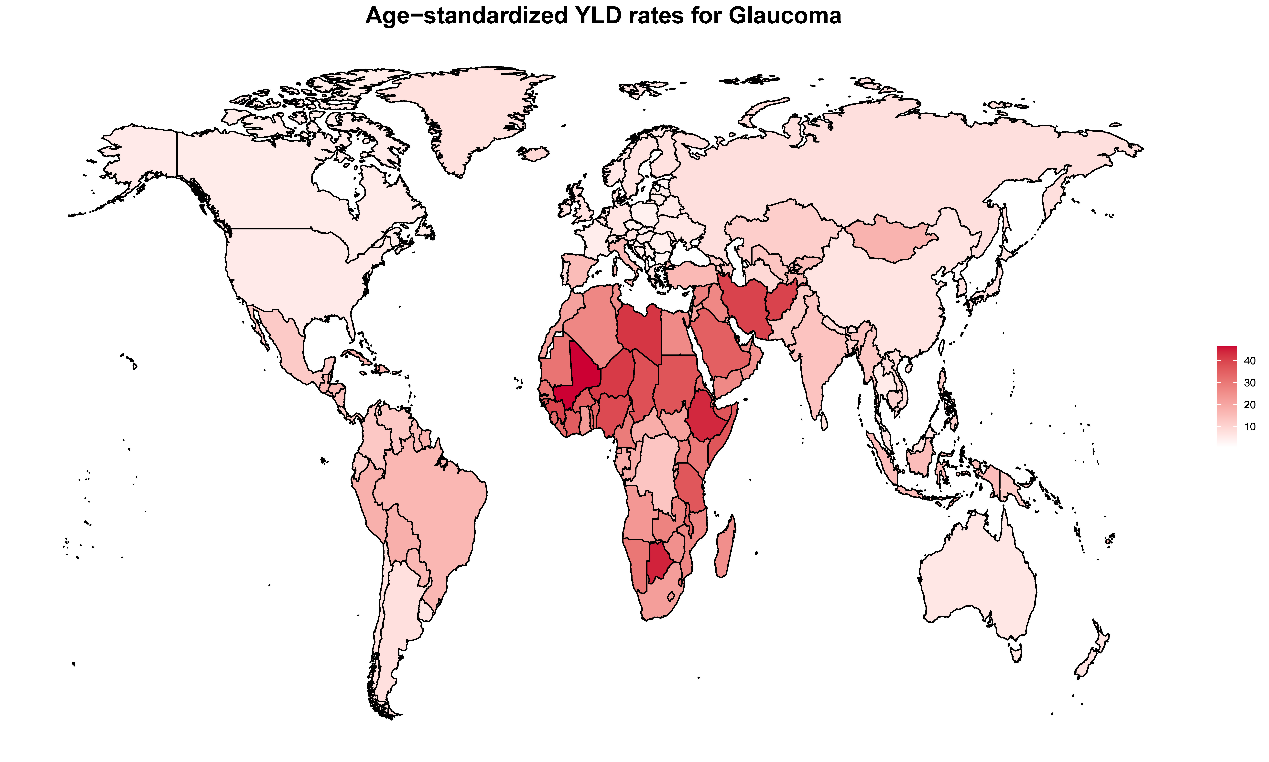


**Supplementary Figure 8**. The age-standardized YLD of blindness and distance vision impairment due to hemolytic disease and other neonatal jaundice in both sexes combined, 2019


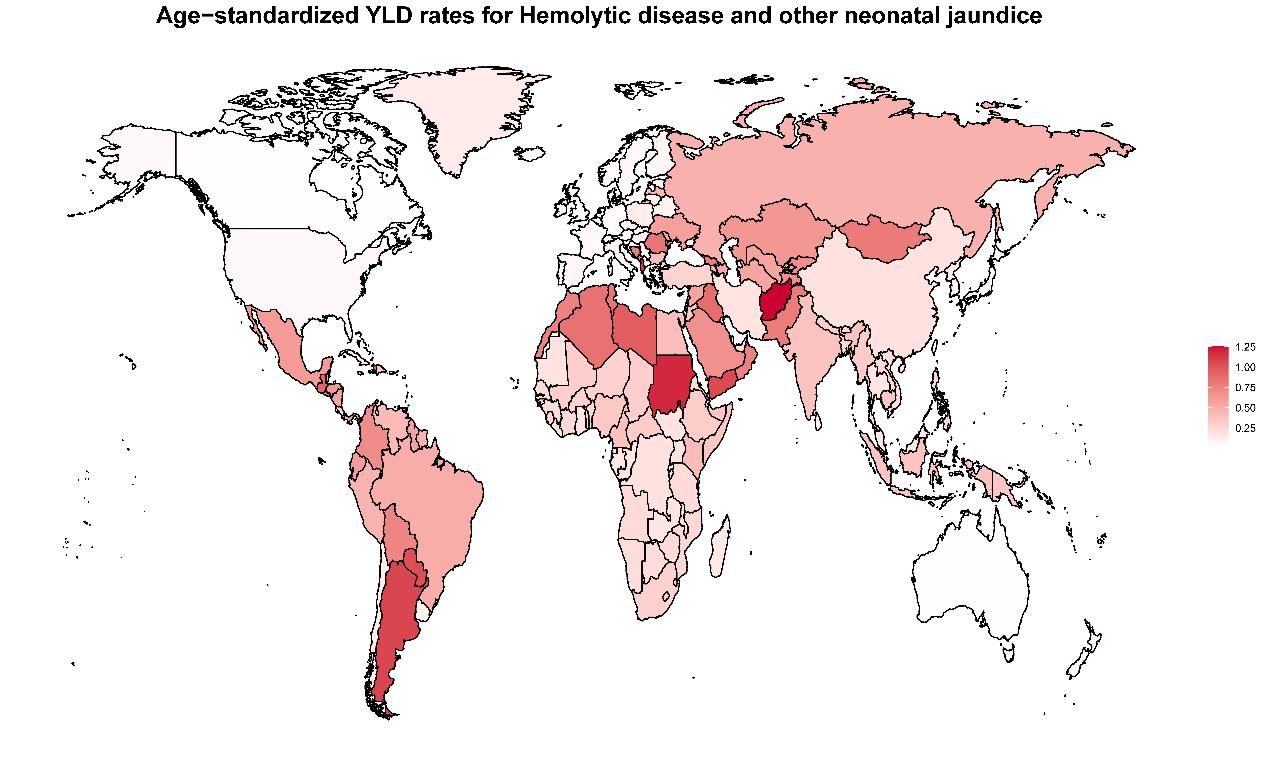


**Supplementary Figure 9**. The age-standardized YLD of blindness and distance vision impairment due to malaria in both sexes combined, 2019


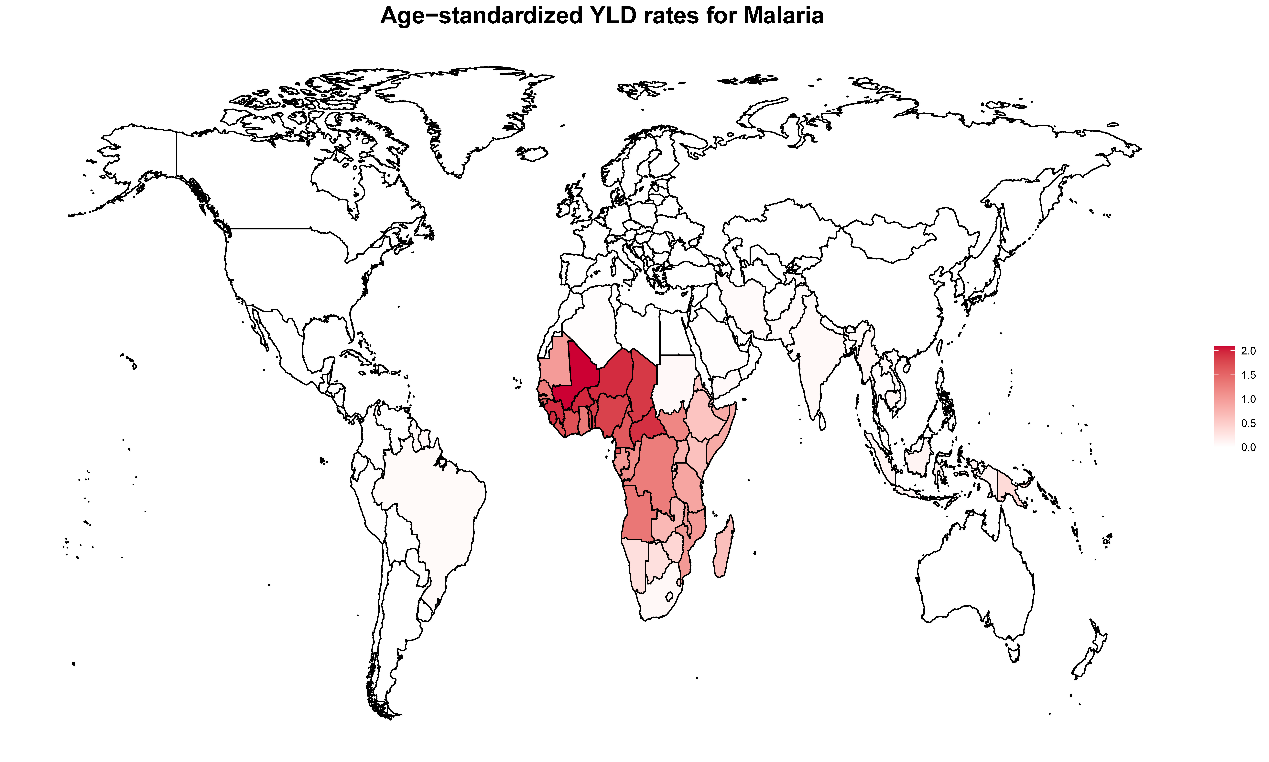


**Supplementary Figure 10**. The age-standardized YLD of blindness and distance vision impairment due to meningitis in both sexes combined, 2019


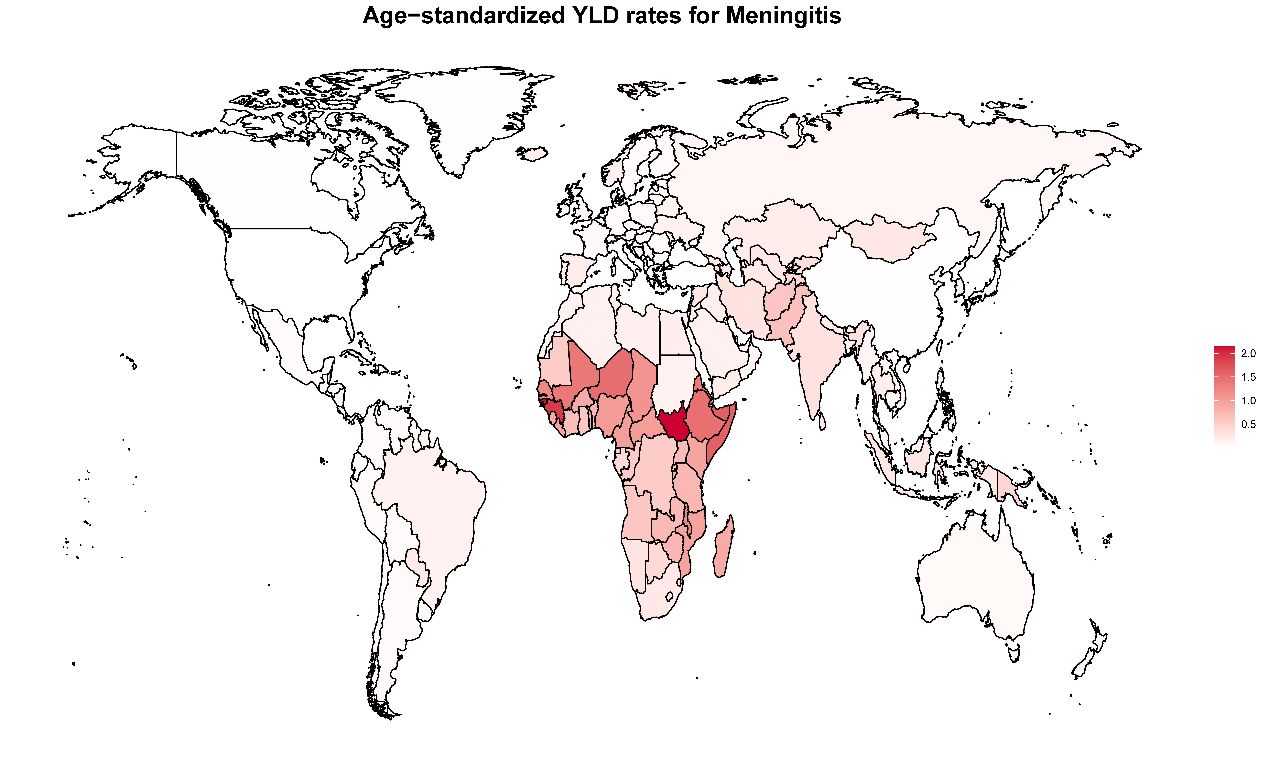


**Supplementary Figure 11**. The age-standardized YLD of blindness and distance vision impairment due to neonatal encephalopathy in both sexes combined, 2019


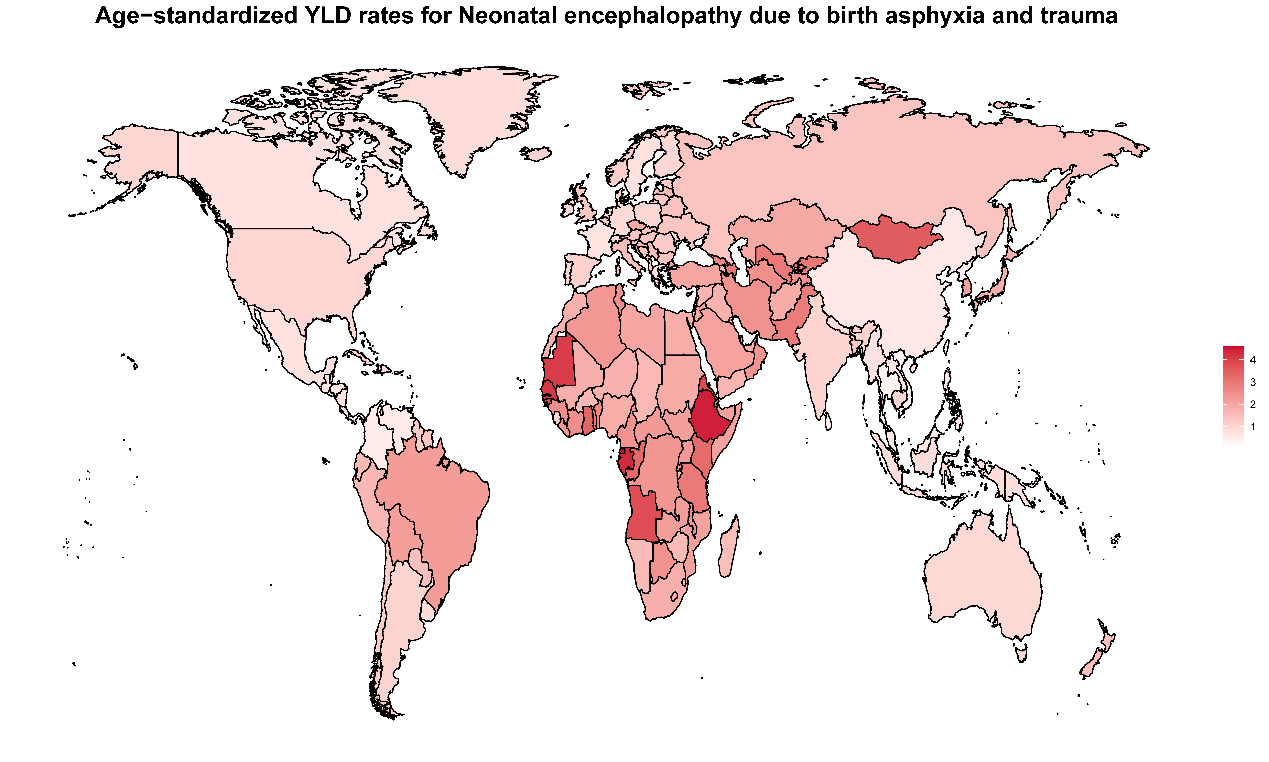


**Supplementary Figure 12**. The age-standardized YLD of blindness and distance vision impairment due to neonatal sepsis and other neonatal infections in both sexes combined, 2019


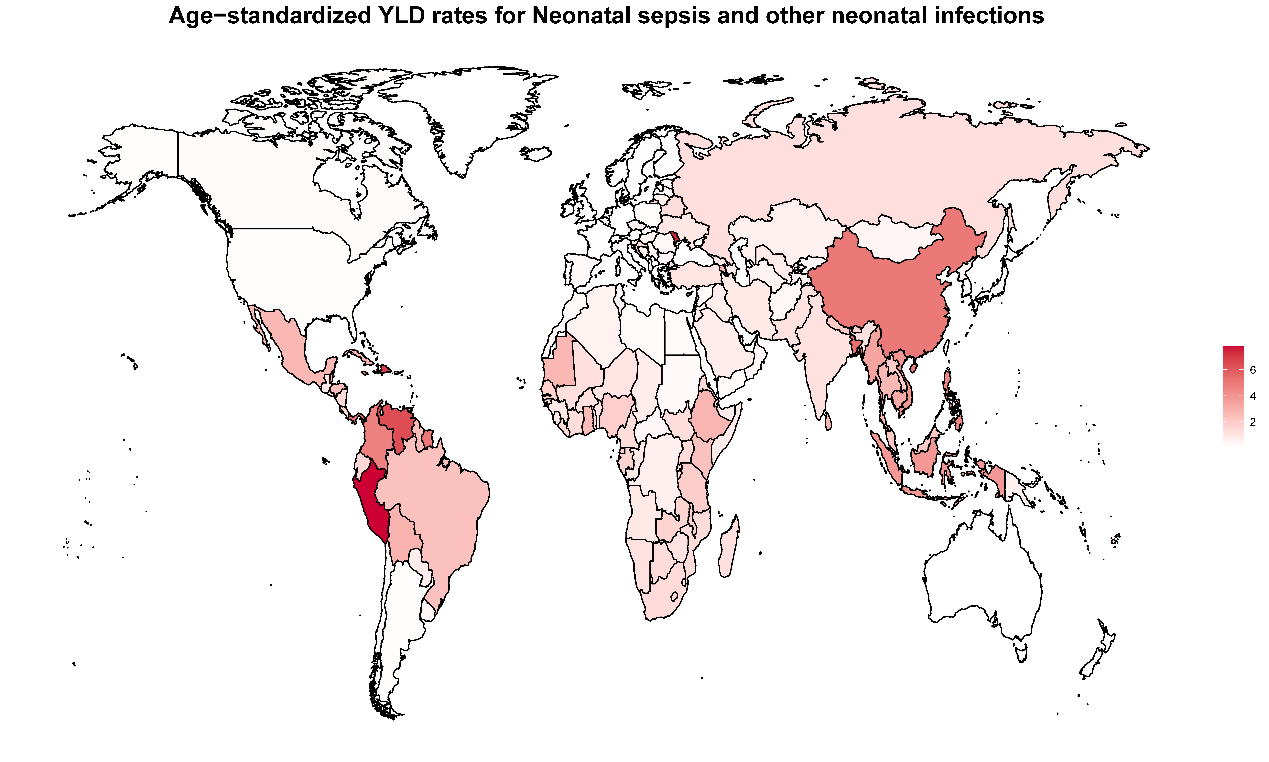


**Supplementary Figure 13**. The age-standardized YLD of blindness and distance vision impairment due to onchocerciasis in both sexes combined, 2019


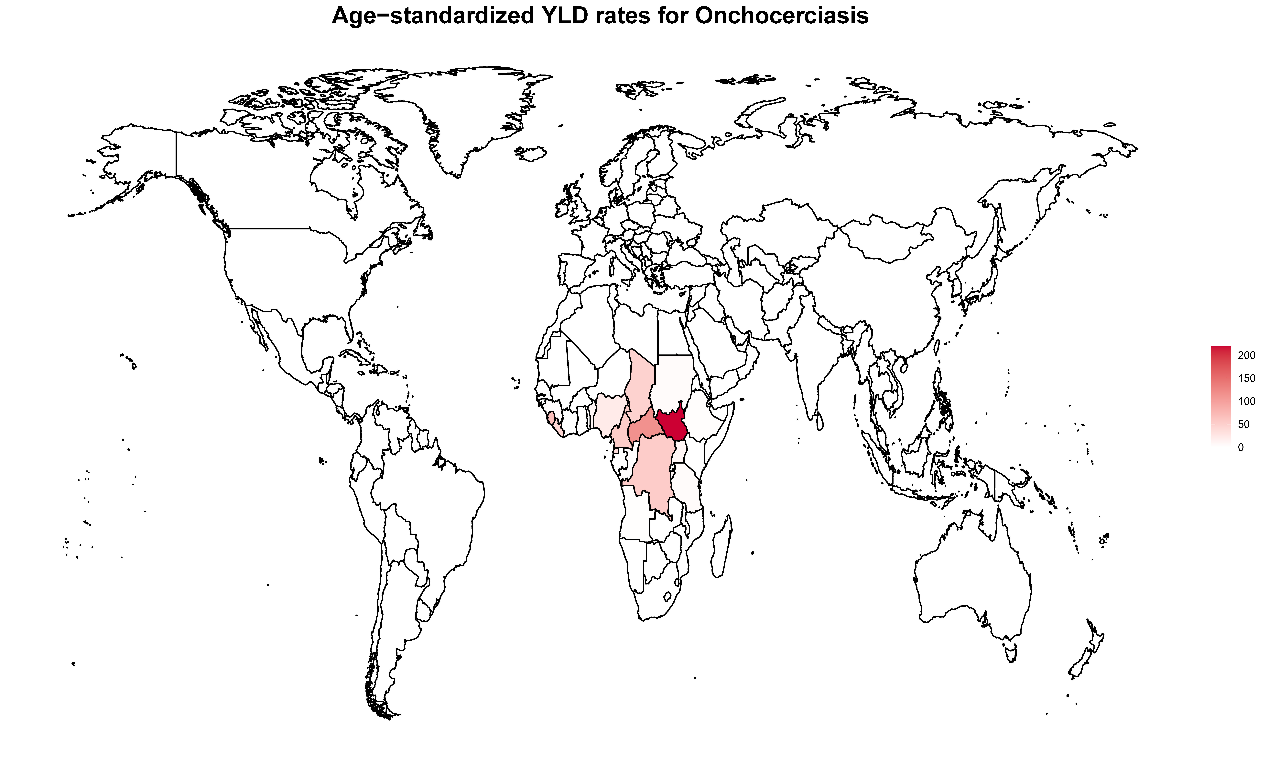


**Supplementary Figure 14**. The age-standardized YLD of blindness and distance vision impairment due to other vision loss in both sexes combined, 2019


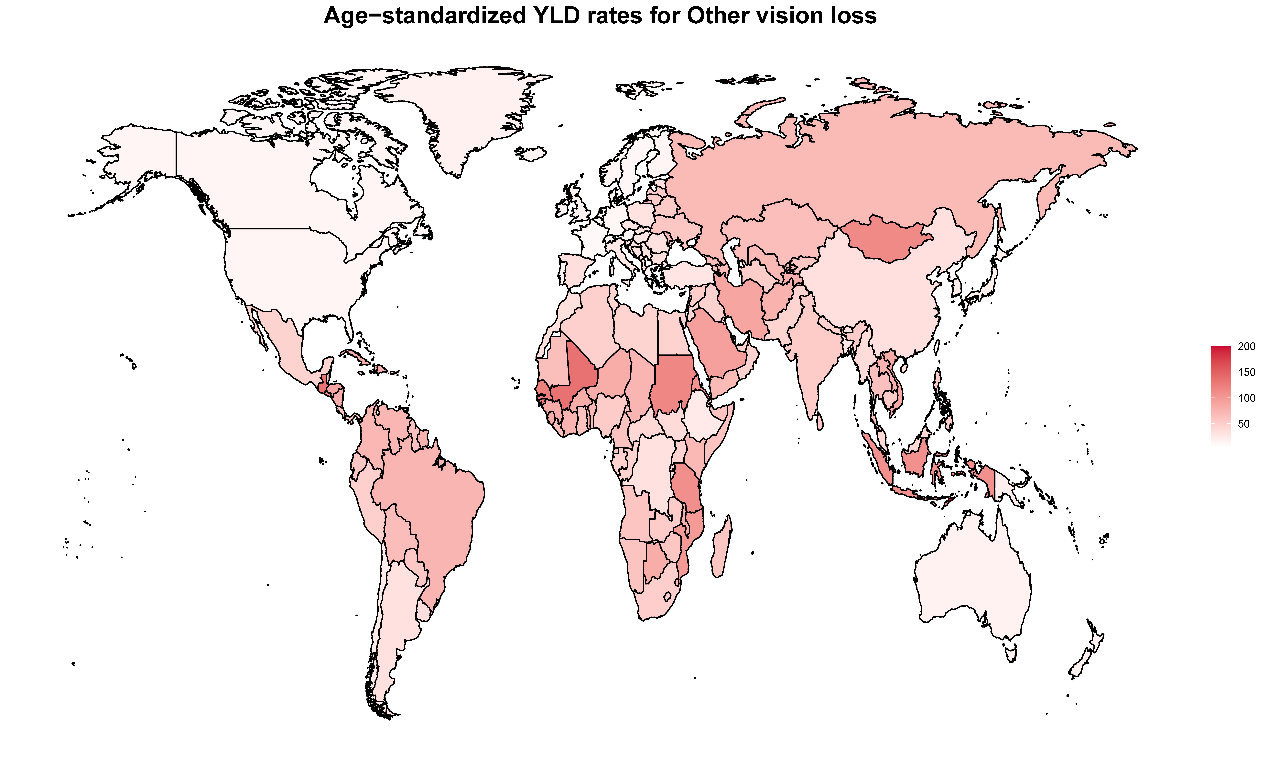


**Supplementary Figure 15**. The age-standardized YLD of blindness and distance vision impairment due to refractive error and other neonatal infections in both sexes combined, 2019


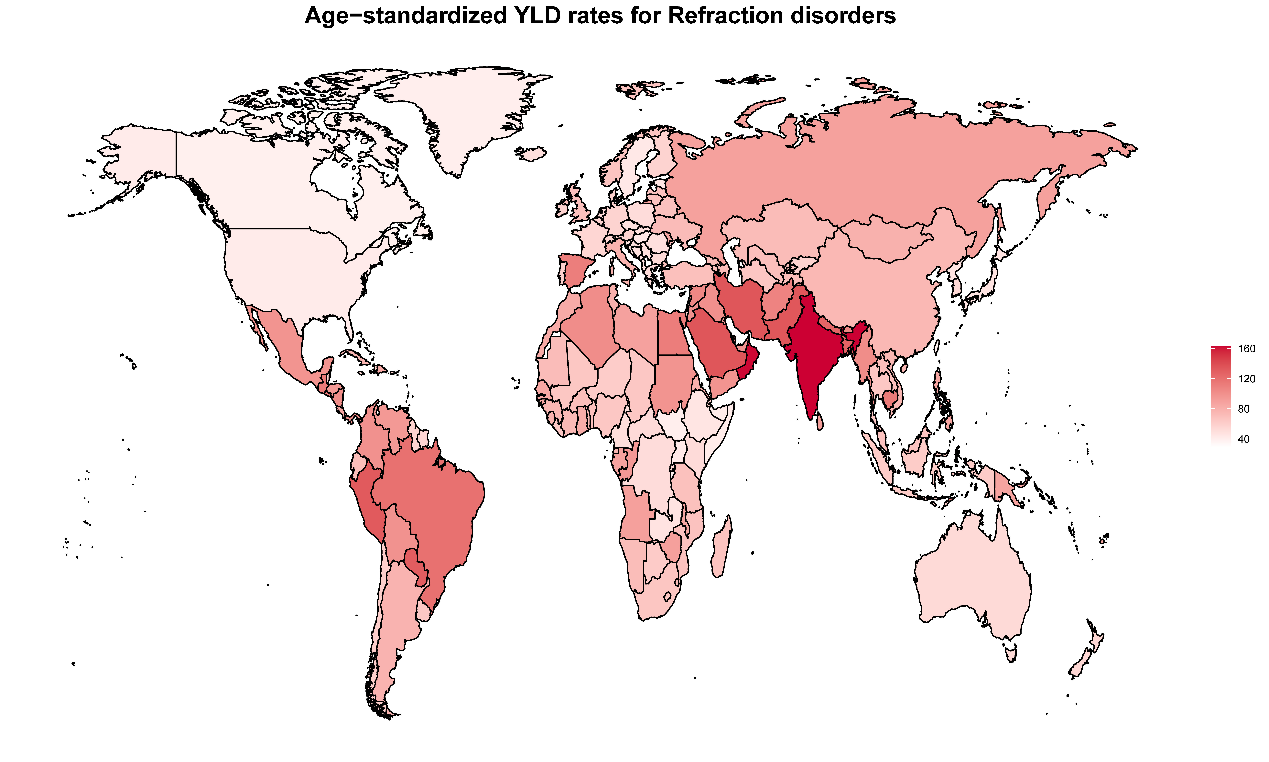


**Supplementary Figure 16**. The age-standardized YLD of blindness and distance vision impairment due to retinopathy of prematurity in both sexes combined, 2019


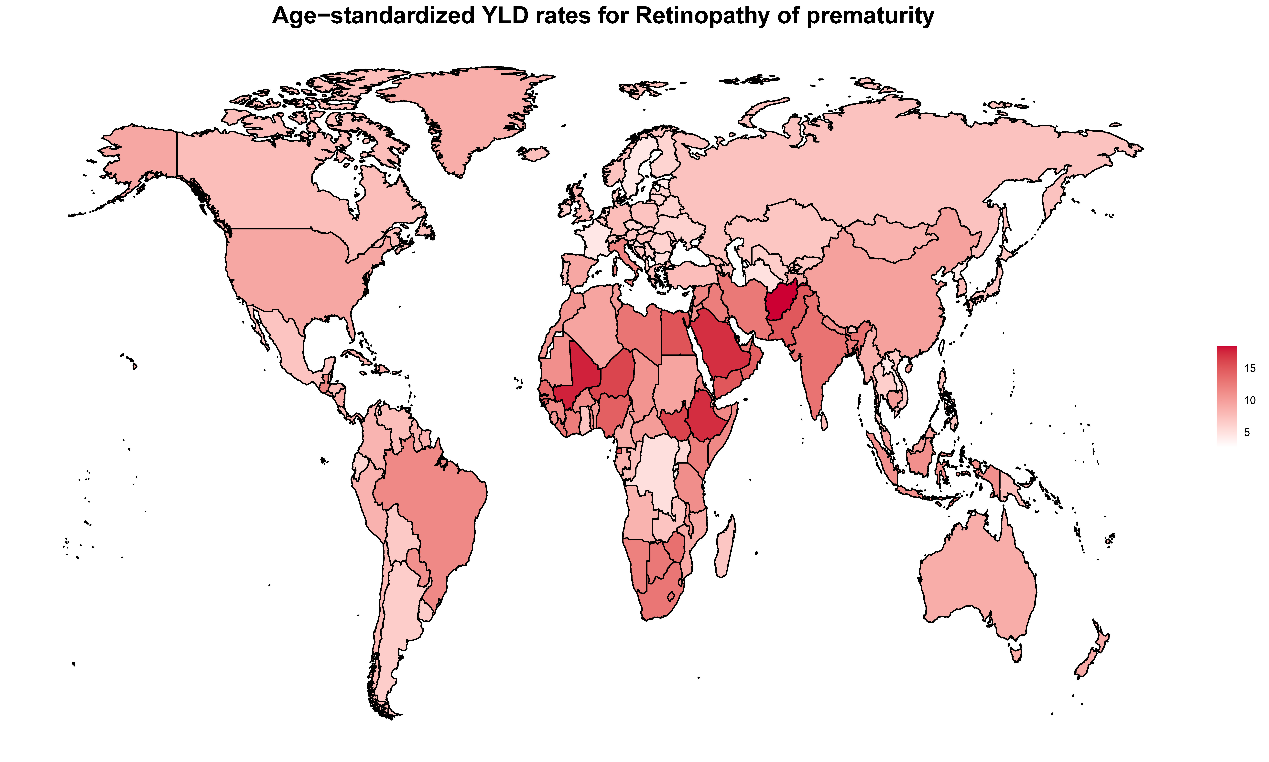


**Supplementary Figure 17**. The age-standardized YLD of blindness and distance vision impairment due to trachoma in both sexes combined, 2019


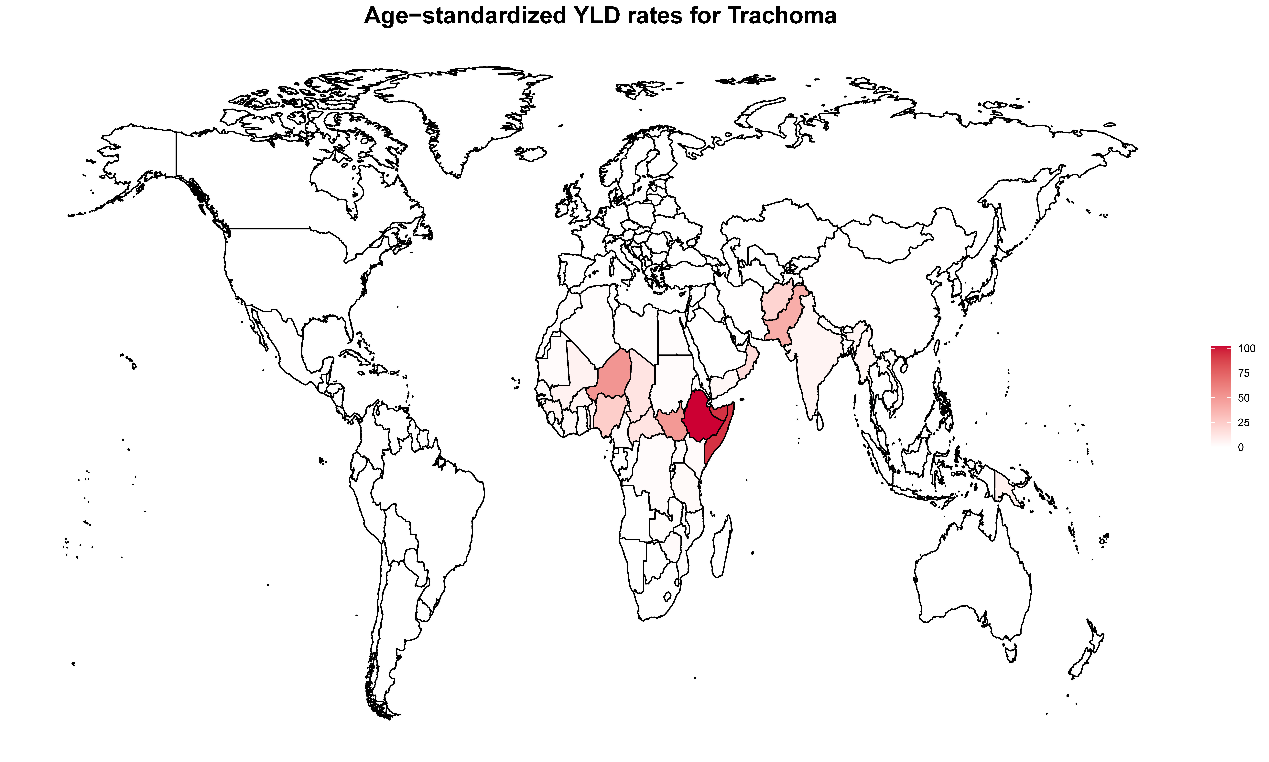


**Supplementary Figure 18**. The age-standardized YLD of blindness and distance vision impairment due to vitamin A deficiency in both sexes combined, 2019


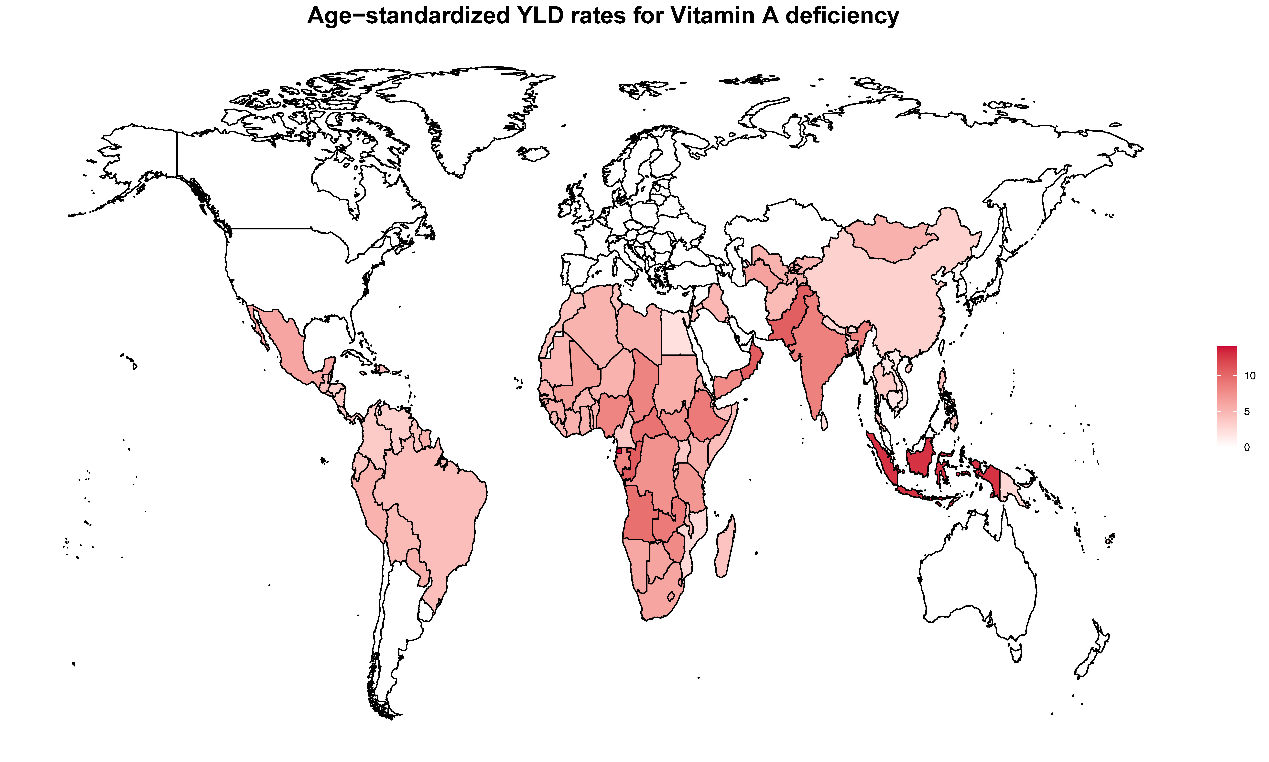


**Supplementary Figure 19**. Cause-specific YLD rates of blindness and distance vision impairment by age groups, 2019


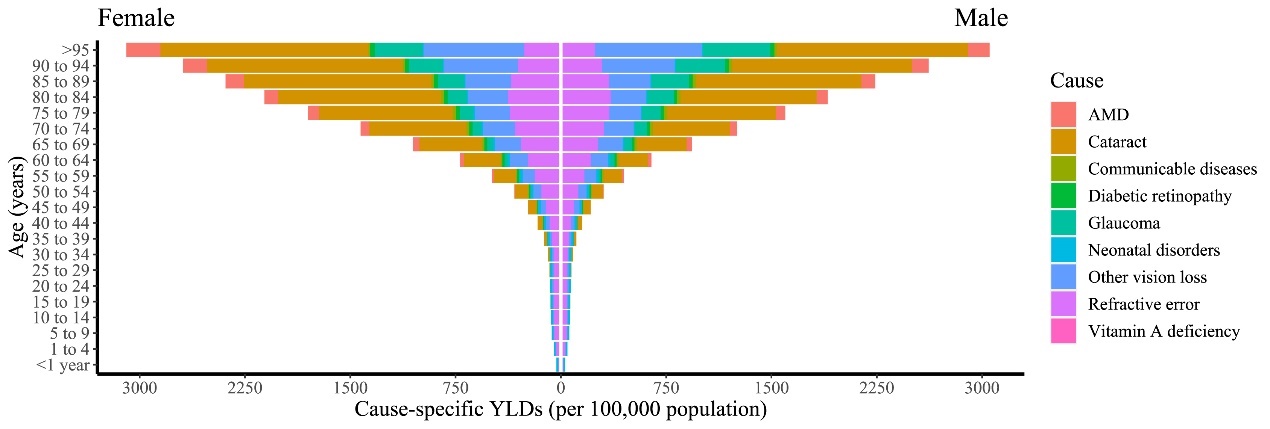


**Supplementary Figure 20.** Cause-specific YLD ratios (female divide by male) of blindness and distance vision impairment, 2019


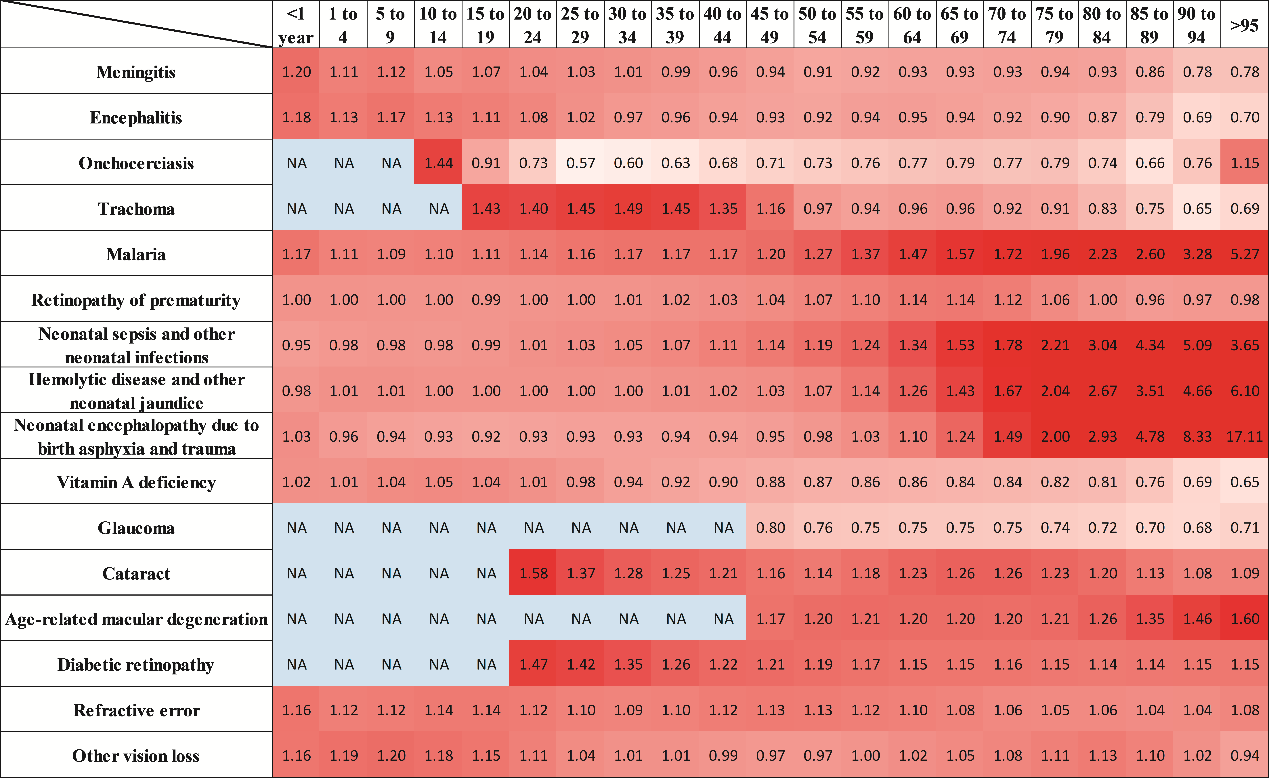


**Supplementary Figure 21.** Cause-specific YLD rate due to communicable diseases by Socio-Demographic Index groups, 2019


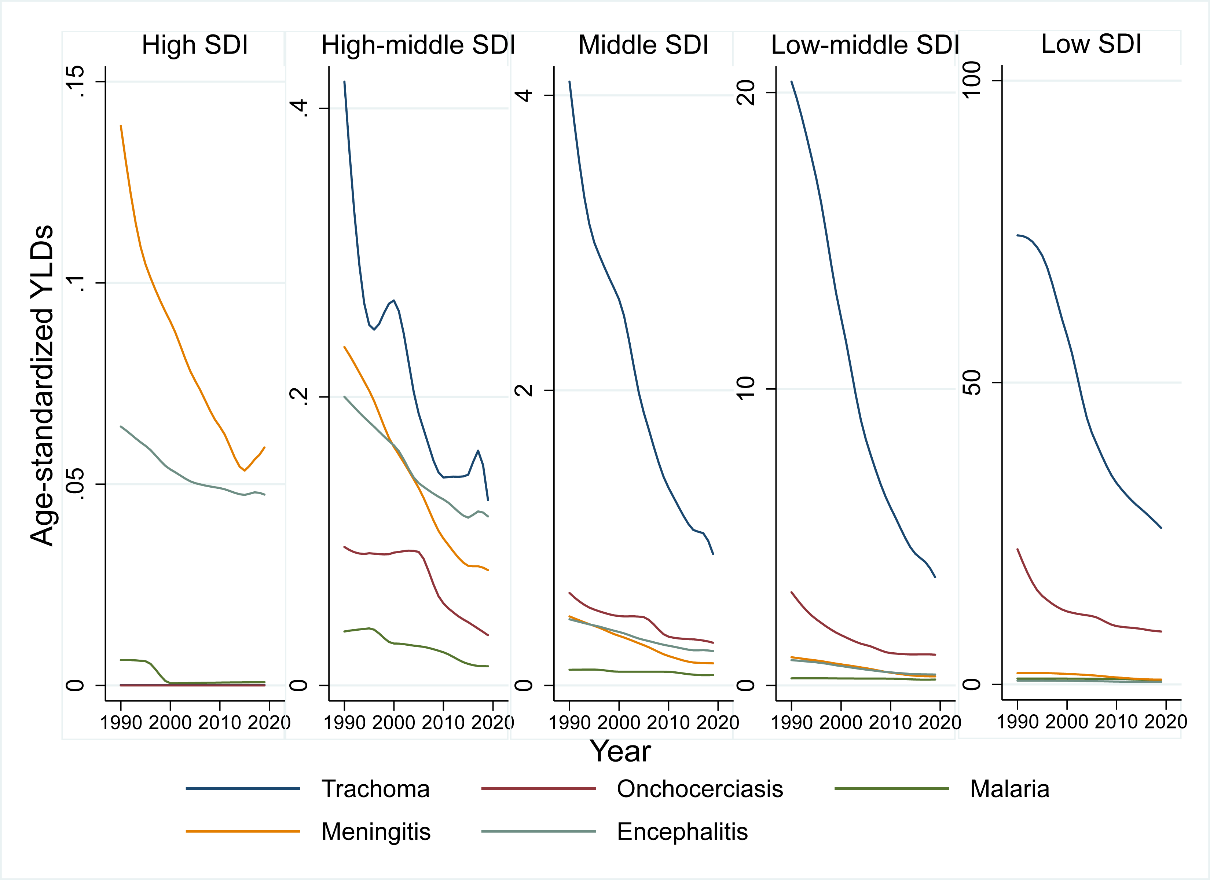

Supplement: Supplementary file 1 [file Table_1.DOCX]
